# Causal inference of gene regulation with subnetwork assembly from genetical genomics data

**DOI:** 10.1093/nar/gkt1277

**Published:** 2013-12-09

**Authors:** Chien-Hua Peng, Yi-Zhi Jiang, An-Shun Tai, Chun-Bin Liu, Shih-Chi Peng, Chun-Ta Liao, Tzu-Chen Yen, Wen-Ping Hsieh

**Affiliations:** ^1^Departments of Resource Center for Clinical Research, Chang Gung Memorial Hospital, Taoyuan 33305, Taiwan, Republic of China, ^2^Institute of Statistics, National Tsing Hua University, Hsinchu 30013, Taiwan, Republic of China, ^3^Nuclear Medicine and Molecular Imaging Center, Chang Gung Memorial Hospital, Taoyuan 33305, Taiwan, Republic of China and ^4^Department of Otorhinolaryngology, Head and Neck Surgery, Chang Gung Memorial Hospital, Taoyuan 33305, Taiwan, Republic of China

## Abstract

Deciphering the causal networks of gene interactions is critical for identifying disease pathways and disease-causing genes. We introduce a method to reconstruct causal networks based on exploring phenotype-specific modules in the human interactome and including the expression quantitative trait loci (eQTLs) that underlie the joint expression variation of each module. Closely associated eQTLs help anchor the orientation of the network. To overcome the inherent computational complexity of causal network reconstruction, we first deduce the local causality of individual subnetworks using the selected eQTLs and module transcripts. These subnetworks are then integrated to infer a global causal network using a random-field ranking method, which was motivated by animal sociology. We demonstrate how effectively the inferred causality restores the regulatory structure of the networks that mediate lymph node metastasis in oral cancer. Network rewiring clearly characterizes the dynamic regulatory systems of distinct disease states. This study is the first to associate an *RXRB*-causal network with increased risks of nodal metastasis, tumor relapse, distant metastases and poor survival for oral cancer. Thus, identifying crucial upstream drivers of a signal cascade can facilitate the discovery of potential biomarkers and effective therapeutic targets.

## INTRODUCTION

To identify disease-causing genes, the genes should be characterized in the context of regulatory systems. Thus, a major goal of research conducted for identifying disease-causing genes is to elucidate the causal interrelationships among DNA, RNA, proteins and metabolites. The regulatory systems underlying diseases can be abstracted into directed gene networks, which potentially provide the cellular context of dysregulated genes in a given disease state. Recent studies have gone beyond discovering differentially expressed genes in diseases and have attempted to infer dysfunctional regulatory gene networks. Directed gene network inference is the task of deciphering the causal relationships among genes. Typically, causal inference requires target perturbations, which can be either experimental perturbations (e.g. single gene knockout) or natural genetic perturbations (e.g. DNA polymorphisms). Natural genetic polymorphisms are widely recognized to facilitate the inference of causations in networks, and current studies have adopted this strategy by using expression quantitative trait loci (eQTLs).

The eQTL mapping process identifies genomic regions responsible for altering the expression of genes across a population. Therefore, eQTL markers can be regarded as natural perturbations that result in distinct gene activities, and these markers can serve as anchors to orient the edges in a gene network (1–3). Consequently, eQTL information has been used to identify causal relationships between complex traits and reconstruct causal gene networks. The eQTL mapping process can help reduce the size of equivalence classes in the possible causal networks that identically explain gene expression profiles (4). Schadt *et al.* (1) proposed the conditional independence test to infer the causal, reactive and independent relationships between 2 genes, and follow-up studies have considered the orientation of each pair of genes in the network (2,5). Structural equation modeling (SEM) is also a popular method for causal inference (6,7); however, most SEM-based models must search large numbers of possible networks, and researchers have attempted to alleviate this problem by using optimization algorithms (8–10). In addition to the aforementioned two-step strategies, joint inference of eQTLs and their corresponding causal networks have been attempted recently (4,11). Neto *et al.* (4) used the Markov chain Monte Carlo (MCMC) method to iteratively update network structure through single-edge proposals and estimate QTLs conditional on the proposed phenotype network. However, this intensive computing task is a bottleneck to the scalability. Hageman *et al.* (11) described another approach to jointly infer a genotype–phenotype map by using Bayesian and improved MCMC strategies. However, this approach can only accommodate a network on the order of 30 nodes.

Most current strategies encounter the same problems of computational efficiency and difficulties in handling genome-wide eQTL data. Therefore, to overcome the efficiency problem in causal network inference, we propose a novel method to deconstruct a global map into multiple subnetworks that integrate phenotype-associated gene modules and their driving eQTLs. The first step toward this goal was to preselect the functional modules related to a phenotype of interest. In subsequent steps, the causality relationships among the module members were inferred by integrating eQTLs. Finally, all the local subnetworks were assembled through a ranking strategy. We present a brief summary of each step and related research next.

In the postgenomic era, a key challenge is to relate the status of a disease with the underlying collective changes in gene activities. Thus, identifying phenotype-associated functional modules is the first step in pinpointing the dysfunctional regulatory systems of a disease. A functional module refers to a set of active genes in this study because genes function in concert rather than independently. Traditionally, coexpression networks have been used to identify functional modules in several diseases (12–15). In this category, WGCNA provides comprehensive functions for analyzing coexpression networks (16). Nevertheless, a coexpression network covers not only direct interactions between genes but also numerous indirect or confounding associations. To reduce indirect and confounding effects, our coexpression analysis is constrained with physical interactions: we consider coexpression patterns of genes encoding physically interacting pairs of molcules. Because the human interactome is growing dramatically in coverage and quality, integrating expression profiles with molecular interaction data enables the detection of previously unknown active modules beyond the scope of well-defined pathways. To address this problem, several methods have been developed to identify differentially expressed modules from the human interactome (17–20). In pioneering work, Ideker and colleagues devised (18) an aggregate z-score and mutual information to select modules that are most associated with phenotypes. Hwang *et al.* (19) proposed a MANOVA-based scoring method to consider the correlation structure of genes; thus, the functional module identified tends to consist of highly correlated genes. Other edge-based approaches detect active modules with the topology structure of condition-relevant interactions (21–23). Most of these methods are based on the well-known hypothesis that the expression profiles of functionally relevant genes are typically highly correlated. By contrast, rewiring a signaling network has recently been shown to induce phenotypic changes in cancer cells and generate disparities in coordinated gene coexpression patterns in distinct patient groups (24,25). Dynamic modularity is suggested to be highly sensitive to physiological conditions and is thus considered to determine tumor phenotypes and patient outcomes. Therefore, we designed a hybrid scoring method by integrating molecular interaction data and gene expression to rank interplay partners and to measure the potential of a functional module for discriminating a phenotype of interest.

Our construction of subnetworks requires the detection of the eQTLs underlying each gene module. Traditional eQTL analysis refers to single-trait analysis or single-marker analysis, which considers one transcript or one marker at a time and requires hundreds of thousands of model fittings (26–29). Thus, both types of eQTL mapping lead to high false-positive rates. Advanced eQTL mapping methods, such as Bayes (30), have combined all the transcripts and markers, but these methods still cannot deal with highly correlated markers in the linkage disequilibrium block. These methods tend to arbitrarily select only one marker out of the set of correlated markers. In fact, most eQTLs do not exhibit strong effects, and most transcripts are usually associated with several loci, particularly physically linked ones with small effects (31). To overcome these challenges, Lirnet regularized the problem by bounding the L1 and L2 norms of the solution and relied on prior biological knowledge about genetic markers, such as sequence conservation, synonymous types, and splicing effects (32). Similarly, in our study, we adopted the sparse partial least squares (SPLS) regression because of its specific advantages. First, SPLS regression reduces the false-positive rate by alleviating multiple testing because the regression considers multiple transcripts as a multivariate response and accommodates tens of thousands of markers at a time using both L1 and L2 penalties (33,34). The correlations among the transcripts are also considered for capturing markers with small effects. Second, SPLS regression accommodates the grouping structure of genomic markers because it selects groups of correlated markers rather than a single marker. The third benefit is low memory requirement and fast convergence; SPLS regression can better handle the correlation among dense markers and thus gain more power on weak linkages. By including the results of the SPLS, the local causality of each subnetwork is further inferred using the Bayesian network with the preselected markers and module transcripts.

The final step in the framework we propose here is the assembly of all the subnetworks, which is challenging because the local interrelationships among genes are likely to be modified in the context of the full-scale network. This problem is similar to reconstructing a structural network for a society with social species (35). In animal societies, the characteristic of one member to regulate other members is called dominance. The dominance relationships within a social group can be arranged into a nonsequential network (35). Numerous mathematical methods have been developed to determine the dominance hierarchy in an animal society based on the observed competitive interactions among the society’s members (36–39). The pairwise competition outcomes carry information about the dominance hierarchy, and this local information can be transferred into a global hierarchy through information transitivity. By comparison, our proposed approach uses the results of local causality testing in the subnetwork as the decisive outcomes of pairwise competitions between social members. Consequently, this study deduced the causal relationships among genes in a global network from local causalities using a social network ranking strategy (38).

Here, we demonstrate the proposed method using a data set from patients with oral squamous cell carcinoma (OSCC). The resulting network was constructed with modules that might function in lymph node metastasis, and the causal inference was compared with the records in the KEGG pathway. When the inferred regulation differs from that in the regular biological system, network motifs can be adopted as biomarkers to suggest the most upstream causes of the disease. In this study, we evaluated the ability of top network-based biomarkers to detect nodal metastasis among OSCC patients and assessed their prognostic significances for tumor relapse and patient survival outcomes.

## MATERIALS AND METHODS

### Overview of causal gene network inference

**Figure 1.**
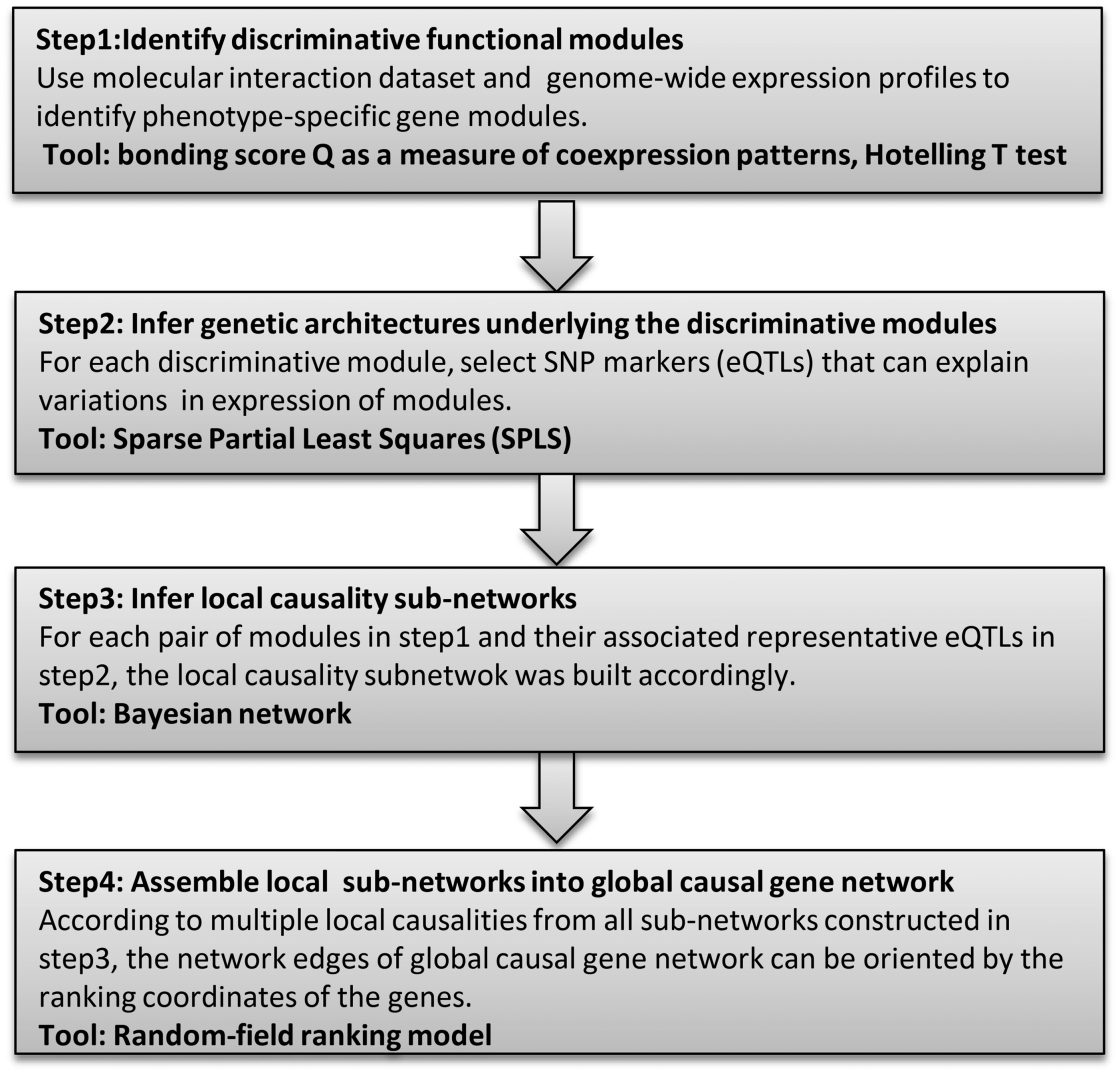
Overview of the proposed method. This flowchart presents a brief overview of the four main steps of causal network reconstruction for a phenotype of interest.

Our goal was to construct a directed network topology representing causal signal flows among genes. We determined the existence of an edge between any given pair of genes and inferred the edge orientation concomitantly. The model space of such a directed network inference may grow at a super-exponential rate with increasing numbers of genes. Thus, to enhance efficiency and scalability, we searched in a ‘divide-and-conquer’ manner. An overview of analysis steps and the rationale behind them is presented next and is summarized in Figure 1.

#### Step 1: Identifying discriminative functional modules for a phenotype of interest

Genes typically cooperate in a modular manner to coordinately carry out specific cellular function. The first step was to identify discriminative functional modules that correlate with distinct phenotypes. In this initial step, gene expression levels were incorporated with a pooled molecular interaction data set that includes protein–protein interactions, protein–gene interactions (40) and known pathways (41). Coexpression patterns of physically interacting pairs were considered for discovering modules.

#### Step 2: Inferring genetic architectures (eQTLs) underlying the discriminative modules

‘Perturbations’ of genes are known to be required for uncovering causal genetic relationships. Naturally occurring genetic variations are one source of such perturbations that can be used to infer causal gene network. Thus, eQTL data can aid in causal inference, where causality follows from the central dogma (i.e. DNA variations lead to changes in gene expression profiles or functional sites in proteins, which in turn alter the activity of other genes or change phenotypes). In brief, this causal nature of genetics allows the orientation of the downstream genes to be anchored under the common eQTLs, thereby providing the roots for the causal network structure. Thus, for each discriminative functional module from Step 1, SPLS regression was used to identify the corresponding eQTL markers that control the expression patterns of the module members in this step.

#### Step 3: Inferring local causality subnetworks

To distinguish disease-causing genes from their downstream responsive genes, we adopted the Bayesian network, whose nodes represent genes or eQTLs and whose directed arcs represent conditional probabilistic dependencies. Such a model is intrinsically capable of depicting the flow of causality among multiple interacting genes (i.e. the presence of a directed edge from node x to node y implies gene x regulates gene y). However, several network structures that represent distinct causal processes are likelihood equivalent. To orient network edges that cannot be determined using expression data alone, we added eQTL nodes into the Bayesian network. This is because an eQTL may have causal effects on the expression of certain genes. Because a gene and its upstream regulators probably share underlying eQTLs, numerous edge directions were precluded based on the central dogma (mentioned in Step 2). Incorporating eQTL nodes can break likelihood equivalence in a Bayesian network by creating new conditional independence relationships and can exclude a large portion of the space of possible networks.

#### Step 4: Assembling local subnetworks into a global causal gene network

In the preceding step, Bayesian network inference was performed for each pair of modules and their corresponding eQTLs. With this approach, the regulation direction for any pair of genes is inferred more than once. Only the counts of regulatory directions were recorded for each pair of genes under all the subnetworks covering the 2 genes. This summarized information was then assembled together with the random-field ranking method to decide all the edge orientations in a global network.

### Single-nucleotide polymorphism typing and gene expression profiling

Genome-wide single-nucleotide polymorphism (SNP) genotypes and gene expressions of 57 OSCC primary tumors were measured by Affymetrix SNP Array 6.0 platform and Exon 1.0 ST array (42). The data were obtained from the Gene Expression Omnibus database under accession number GSE25104. Among the 57 OSCC samples, 38 nodal metastasis cases were pathologically confirmed. The remaining 19 cases were free of nodal metastasis for at least 36 months.

### Identification of discriminative modules

A module is defined as a gene set whose members have interactive interrelationships in the human interactome (40,41). Each gene in the molecular interaction data can be a seed for a module candidate. To expand the module iteratively from a seed gene, a hybrid scoring method was used to prioritize the inclusion of interplay neighbors of a seed. This hybrid scoring method combined 2 types of scoring rules. First, because functionally collaborative genes tend to exhibit highly correlated expression patterns (43,44), for each physical interaction *i* between the current module member *x_i1_* and its interacting neighbor *x_i2_* outside the module, we defined

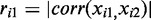

where *corr* is the Pearson correlation coefficient (PCC) of gene expression between *x_i1_* and *x_i2_* that is calculated from all samples to evaluate their cooperative potentials.

Second, dynamic changes in the organization of the human interactome have been demonstrated to be associated with disease outcomes (24). The dynamic genes may at least partly organize the communication of functional pathways, and their expression profiles might positively correlate under one condition and negatively correlate or not correlate under another condition. Thus, we defined the following measure of correlation difference:

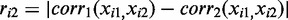

where *corr*_1_ is the PCC of gene expression between *x_i1_* and *x_i2_* among the samples for one condition, and *corr*_2_ is the PCC of gene expression between *x_i1_* and *x_i2_* among the samples for the other condition.

We calculated r_i1_ and r_i2_ for each physical interaction *i*. To evaluate the magnitude of the correlation r_i1_ and the correlation difference r_i2_, we further ranked either numbers, respectively, among all physical interactions in the human interactome data set. Accordingly, each physical interaction received 2 percentiles, q_i1_ and q_i2_. Thus, a bonding score is defined as follows:

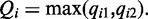



A physical interaction with a high bonding score could indicate that the corresponding pair of genes correlates highly across all samples or displays a large disparity in correlation between 2 sample groups. Therefore, we regard the interaction to be active if its bonding score is sufficiently high. Consequently, a neighbor of the current module members was considered a candidate for entry into the module if its binding score was higher than that of other neighbors and greater than a prespecified *r*. If the highest bonding score was less than *r*, the module stopped expanding. Because we sought only potentially active interactions, we considered *r* = 90% in this study to exclude physical interactions with bonding score below the 90^th^ percentile. Furthermore, we tested the ability to discriminating disease status with the joint expression profiles by using the Hotelling’s T test when the candidate was included as a module member. If passed a significance level of 0.05, this neighboring gene was included in the expanded module. The neighbors of the new member were considered for inclusion in the next iteration. The iterative expansion procedure was stopped when the addition of any neighboring genes did not pass the Hotelling’s T test, or when the size of the expanded module exceeded 10. The module size was limited to 10 because a large module seldom has common driving eQTLs that are useful for anchoring network edges. Furthermore, in the proposed method, we assembled small Bayesian subnetworks to reduce the intrinsic heavy load of directly inferring an entire large network. With increasing module size, the time complexity for inferring a single Bayesian subnetwork in Step 3 grows exponentially (Supplementary Figure S1), which diminishes the benefit of assembling subnetworks. As Supplementary Figure S1 indicates, the time required for a module size of 15 is nearly double that for a module size of 10, and the inference time increase enormously thereafter. Hence, to efficiently construct subnetworks for each pair of modules, 10 is a feasible limit of module size. The Hotelling’s T test procedures are described in detail in Supplementary Methods.

### Mapping the driving eQTLs underlying discriminative modules

The second step was to identify sequence variations that potentially cause alterations in the expression of discriminative modules. We attempted to perform module-level eQTL mapping. The transcripts within each module were considered as multivariate response variables, and SNPs were considered as predictors. However, the ordinary least squares regression model cannot handle a multivariate response variable and a large number of SNP markers, especially for the ‘small n, large p’ problem. Even the general multivariate regression model does not handle the ‘n<<p’ problem and the collinearity among explanatory variables. Regularized methods have attempted to overcome the problem by bounding the L1 or L2 penalty. The LASSO is one such method. Although using LASSO assures sparseness of the solution, the number of variables selected by the LASSO is bounded by the number of samples. The LASSO also tends to arbitrarily select one variable out of several highly correlated covariates. To overcome these limitations for multivariate responses, we adopted the SPLS regression, which was developed based on solid theoretical support (33,34). The SPLS regression achieves variable selection by using the L1 penalty to set the coefficients of the irrelevant variables to 0. The SPLS regression also performs grouped selection by imposing the L2 penalty to handle multicollinearity among covariates, which results in groups of collinear variables (34). Thus, the SPLS regression avoids the curse of dimensionality and can select the entire group of correlated variables into the model without a predefined grouping structure. The problems solved by the SPLS regression were also combated by the elastic net described by Zou and Hastie (45). The elastic net handles multicollinearity in the variable selection problem by combining the LASSO with ridge regression. However, SPLS regression copes with multicollinearity by integrating Wold’s PLS algorithm (46), which is based on basic latent decomposition and conjugate gradient that accelerate its convergence and reduce its storage demand. Because the algorithm of SPLS was designed to achieve better efficiency and require lower memory usage than the elastic net, we used SPLS regression for selecting eQTLs.

We considered the module-level eQTL model

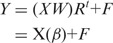

where the matrices *Y* and *X* represent the *q* response variables and *P* explanatory variables, respectively, in *n* samples; *W* transforms *X* into uncorrelated variables; the matrix *R* is a coefficient matrix; and *F* is an error matrix. Both columns of *Y* and *X* are assumed to be centered to zero. To find the latent variables that have maximum covariance with the response variables, the SPLS model derives the direction vectors in the columns of *W* by adding an *L*_1_ and *L*_2_ penalties:

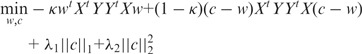

restricted to 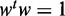 and *c* is estimated a priori as

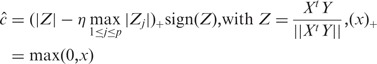



The numbers *η* and *κ* are two parameters to control the size of the final model. Because of the sparsity of matrix *W*, most of the estimates for the coefficient *β* are zeros and it leads to selection of variables in our eQTL mapping process.

The model building algorithm was implemented in the R package called *spls* and used to map the associated SNPs that control the expression of the discriminative modules. Because cis-acting elements (cis-markers) of gene expression usually acquire major effects (47), we tested the association between a given module and all of the SNPs within 1 million bases of the transcription start or stop sites of all corresponding module members. Because most module members were derived from distinct chromosomes or were located far apart on the same chromosome, this set of cis-markers for any gene could be trans-acting elements (trans-markers) for the remaining members. Therefore, cis-markers and trans-markers from functionally related genes within the same module were simultaneously analyzed. We typed the SNP data by using high-density Affymetrix SNP 6.0 arrays, and the size of SNPs was still a challenge for the SPLS model. Thus, we adopted a two-step strategy to screen eQTLs for each module. In the first step, we perform SNP selection on each chromosome, and in the second step, we combined all the selected SNPs to build an overall model by using SPLS regression again.

As described by Hageman *et al.* (11), highly linked SNPs change a network’s topology. Therefore, we adopted a heuristic rule to screen for unlinked representative SNPs. Unlike the LASSO that arbitrarily chooses one SNP from a group of physically or genetically linked SNPs and ignores the remaining SNPs, the SPLS regression captures all correlated SNPs with a grouping structure, which allowed us to identify the most representative SNP with the strongest effect from the entire group of correlated SNPs. For this purpose, all the SNPs underlying the same module were first grouped into SNP blocks using hierarchical clustering with complete linkage cut at distance of 500 kb, which is commonly assigned as the size of a linkage block (48). For each block, the representative SNP with the strongest association with the module expression was selected using the MANOVA test.

### Bayesian network model for subnetwork inference

The discriminative modules and the underlying eQTLs detected in the previous steps were the building blocks used to construct the network.

Because a Bayesian network describes conditional independence relationships and models the flow of causality among multiple interacting quantities, the mathematical representation of a Bayesian network is similar to that of a causal network. Therefore, we used the framework suggested by Bøttcher and Dethlefsen to compute a network score with the joint multivariate probability distribution (49). The eQTLs can be upstream causes of altered expression in the Bayesian network, but the reverse regulatory direction from gene to eQTL is not allowed. No connections are allowed between any eQTLs, whereas any regulatory relations between genes are possible. We assumed an additive genetic effect and encoded the genotypes as 0, 1 or 2, but also allowed alternative marker coding. The Bayesian network is described as follows. Node *s* in graph *G* corresponds to the continuous random variable *X*_s_. A directed edge from node *s* to node *t* indicates that *X*_t_ is causally dependent on *X*_s_. Graph *G* follows the Markov assumption, which states that every variable *X_i_* is independent of its nondescendant variables when *Xi* is conditioned on its parent variables in *G*. Thereafter, the joint distribution of *X_i_* that characterizes the network structure can be written in the following product form:
(1)
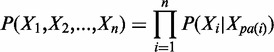


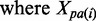
 indicates the variables representing all the parent nodes of *i*.

Given a training data set *D*, the Bayesian network attempts to determine the best-fitted network that maximizes the following marginal likelihood 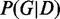:
(2)
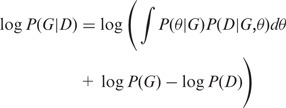

where 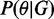 is the prior of the parameters for a given network structure *G*, and *P(D)* is independent of *G*. The marginal likelihood is decomposed according to (1). Therefore, the contribution to the marginal likelihood of each node only depends on the node itself and its parent nodes.

Even with an efficient heuristic searching algorithm, the Bayesian network still has an intensive computational load when the number of nodes is large. Based on the idea of ‘divide and conquer’, the proposed method first teases apart the global network into small overlapping local subnetworks, which expedites the tackling of this problem by using the Bayesian network approach, and then assembles the subnetworks by using a ranking strategy, as described in the next section. Thus, we first build a local causality subnetwork for all genes and eQTLs in each pair of modules. Notably, module memberships or physical interactions among module members are not included in Bayesian network analysis. The regulatory direction between any 2 genes is evaluated relative to their linkage strength with the eQTLs and relative to their expression correlation to the other genes in the Bayesian network. If the signal is robust, the same regulatory direction will be repeatedly inferred, regardless of what their accompanying elements are in the Bayesian inference. This step breaks the boundary of a module incrementally and includes new relationships in addition to the previously known physical interactions through the accumulation of evidence.

Reconstruction of multiple local subnetworks is computationally efficient and can be performed in parallel. Given *n* modules from the previous step, the Bayesian network can construct 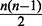 local gene subnetworks. Therefore, the directionality of any pair of genes may be evaluated more than once in the context of distinct module combinations. For example, consider that gene *i* and gene *j* are in the same module; the causal relationship between gene *i* and *j* are evaluated by Bayesian network at least *n* − 1 times, and not all of the results are consistent. Accordingly, the results of multiple local causality tests between any two genes is recorded in an N-by-N causation matrix *C* = [*c_ij_*], i, j = 1, … ,N where 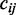 indicates the number of times the i^th^ gene is identified as a direct upstream regulator of the 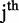 gene, and the N is the total number of genes. The simplest approach to determine the edge orientation is to select the major direction of the corresponding pair of genes: if *C_ij_* is larger than *C_ji_*, the edge is oriented from gene *i* to gene *j*, and vice versa; however, if *C_ij_* equals *C_ji_*, the major directionality cannot be determined and the edge is not created. Other than including this simple decision step that only considers the local information from a pair of genes, we adopted an improved strategy that balances the relative strength of other gene pairs. The random-field ranking method is summarized in the following subsection.

### Random-field ranking procedure for local gene network assembly

A common feature of animal societies is the dominance hierarchy among the social members. This hierarchy resembles the regulatory relationships among genes. Inferring dominance relations among members of a society has been studied in social network analysis. Based on decisive outcomes of pairwise competitions between group members, Fushing *et al.* (38) proposed a random-field ranking method that transforms local dominance relationships into a global nonsequential ranking network through information transitivity. Transitive dominance is indirect information that is computed based on the common interacting partners between two individuals. Because the causation matrix *C* is analogous to local pairwise competitive outcomes in social network analysis, we adopted the random-field ranking method to assemble local causalities of multiple subnetworks.

In the first step of the ranking procedure, the control potentials were estimated based on the causation matrix *C*. A Beta random field comprising random variables *P_ij_* with posterior Beta distribution 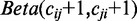 was constructed to infer control potential *P_ij_* for any pair of genes (*i*, *j*) that were based on causation records *c_ij_* and *c_ji_*. *P_ij_* represents the probability that the *i*^th^ gene directly alters the activity of the *j*^th^ gene, and its reverse control potential *P_ji_* equals 1 − *P_ij._* This control probability was converted to direct control odds 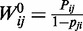. The key feature of this algorithm is the incorporation of indirect dominance information. To consider indirect causal information, a suitable *R*, which is the number of intermediate genes along a path from gene *i* to gene *j*, should be selected. In this study, *R* was only considered to be 1. The transitive control odds were defined as 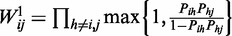, where gene *h* is any common interacting neighbor between genes *i* and *j*. To obtain transitive control odds >1, the product *P_ih_P_hj_* should be sufficiently large. Consequently, weak transitive causal information is not followed. Based on the direct pairwise causalities and transitivity relationships among the additional intermediate genes, matrix *W* of the overall control odds between the *i*^th^ and *j*^th^ genes was defined as

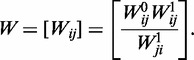

Enhanced control potential matrix P*, based on *W*, was derived as

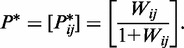


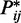
 represents the enhanced probability that gene *i* regulates gene *j*.

In the second step, we used simulated annealing to estimate ranking coordinates of all genes in the global network by minimizing the error accumulation in the ranking protocol (see detailed descriptions in Supplementary Methods). For each gene pair (*i*, *j*), *K* control potential matrices 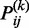 were generated from 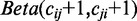. Subsequently, enhanced control potential matrix 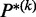 was calculated as mentioned above. Because the values in the lower triangle of any 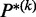 represent the probabilities of lower-ranking genes regulating higher-ranking genes, an error occurs when an element in the lower triangle exceeds 0.5. Consequently, the rows and columns of 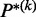 must be rearranged according to an optimal permutation of the ranks of the genes to minimize the number of entries that are >0.5 in the lower triangle. To obtain optimal ranking coordinates for all genes, we followed the method of Fushing *et al.* and used simulated annealing to determine the optimal order (see detailed descriptions in Supplementary Methods). To estimate the relative ranks of the genes in the causation matrix, 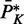 was defined as follows:

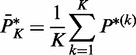



Correspondingly, the global causal gene network was reconstructed from multiple local causalities. If the values of both *c_ij_* and *c_ji_* in the causation matrix were zero, no direct causal relationship was inferred for gene *i* and gene *j* in any local subnetwork. From this perspective, each nonzero component *c_ij_*, 1≦ *i, j* ≦ *N*, in the upper triangle of the causation matrix *C* implied an edge of the global causal network. The network edge was oriented by the relative ranks of the corresponding pair of genes *(i, j)*: gene *j* was causally connected to gene *i* if gene *i* was ranked higher than gene *j*, and vice versa. Using this approach, the global causal gene network can be inferred, as described in ‘Results’ section.

### Model availability

All described methods were implemented in R package. Our package ‘Gemonet’ is hosted at http://www.stat.nthu.edu.tw/∼wphsieh/causalinference.html. The package does not include the random-field ranking algorithm. The original version of the ranking strategy for social networks is available on request through Dr Fushing Hsieh at fhsieh@ucdavis.edu.

## RESULTS

### Identification of discriminative functional modules and their biological significance

Identifying differentially expressed modules is the first step in reconstructing causal gene networks, which are dysfunctional in the lymph node metastasis in OSCC. We overlaid the expression values on corresponding genes in the interaction map and searched for responsive modules that were highly discriminative of lymph node metastasis (see ‘Materials and Methods’ section). Each gene in the interaction data functioned as a seed for expanding a module. A bonding score was defined for each interaction to select genes that are coexpressed or genes that switch their correlation structure between patient groups with and without nodal metastasis. As detailed in Supplementary Methods, the Hotelling’s T test was used to select modules with discriminative power in separate patients groups. This process identified 574 overlapping modules for lymph node metastasis in OSCC, with an average module size of 8.84 genes and a total of 1808 unique genes.

Cancer metastasis is a multistep process that includes the degradation of the extracellular matrix (ECM), apoptosis evasion, cell invasion, migration, angiogenesis, cell adhesion and growth. We performed enrichment analysis on the genes of those 574 modules with DAVID (50,51) to investigate how well the modules functioned in a metastasis-related process, as annotated by Gene Ontology (GO) and KEGG database (41,52). Table 1 lists the 10 most significant GO terms in the category of ‘biological process’, including the regulation of cell proliferation, regulation of apoptosis, cell migration and cell motion, which are recognized as major metastatic processes.

**Table 1.** Top 10 most enriched GO terms in the category of ‘biological process’

GO term	*P*	Bonferroni correction
GO:0010033 ∼ response to organic substance	3.77 × 10^−34^	1.73 × 10^−30^
GO:0009719 ∼ response to endogenous stimulus	3.76 × 10^−28^	1.73 × 10^−24^
GO:0042127 ∼ regulation of cell proliferation	5.82 × 10^−27^	2.68 × 10^−23^
GO:0042981 ∼ regulation of apoptosis	1.24 × 10^−26^	5.71 × 10^−23^
GO:0043067 ∼ regulation of programmed cell death	1.65 × 10^−26^	7.61 × 10^−23^
GO:0010941 ∼ regulation of cell death	2.67 × 10^−26^	1.23 × 10^−22^
GO:0008284 ∼ positive regulation of cell proliferation	1.02 × 10^−20^	4.70 × 10^−17^
GO:0016477 ∼ cell migration	8.27 × 10^−20^	3.81 × 10^−16^
GO:0006928 ∼ cell motion	4.96 × 10^−19^	2.28 × 10^−15^
GO:0051270 ∼ regulation of cell motion	2.89 × 10^−18^	1.33 × 10^−14^

Certain genes appeared repeatedly in the modules, and these were related to tumor metastasis. CYP epoxygenases, which can promote metastasis through CD44-mediated adhesion and CD82 repression, appeared in 22 modules (53,54). The transforming growth factor beta family and SMADs genes, which are required for invasion and prometastatic activities (55), were present in 17 and 9 modules, respectively. Most modules included other key families of genes, such as those encoding ECM and cell adhesion molecules (CAMs); ECM–CAM interactions have been shown to markedly enhance metastatic processes. In addition to the genes directly involved in metastasis, other genes belonging to related processes are recruited in tumor metastasis (23). For example, several modules contained ERBB family genes, cell cycle regulatory genes and the HGF gene for lymphangiogenesis regulation.

Moreover, most members of the 574 modules function in common pathways. Table 2 lists the signaling and metabolic pathways that were significantly enriched. The top pathway is focal adhesion, which is known to play pivotal roles in cancer metastasis. Beyond anchoring the cell, the focal adhesion complex functions as a signal carrier that transmits signals from the ECM. ECM–tumor cell interactions, which rank third in Table 2, are mediated by integrins and are critical in the metastasis cascade. This process triggers signal transduction and increases the tyrosine phosphorylation of focal adhesion kinase (FAK), thereby inducing cell proliferation, survival, migration, invasion and metastasis (56–58). Collectively, the pathways listed in Table 2 contribute to the major events of the invasion–metastasis cascade.

**Table 2.** Significantly enriched KEGG pathways

Pathway in KEGG	*P*	Bonferroni
hsa04510:Focal adhesion	8.22 × 10^−39^	1.57 × 10^−36^
hsa05200:Pathways in cancer	5.05 × 10^−22^	9.64 × 10^−20^
hsa04512:ECM-receptor interaction	1.26 × 10^−21^	2.40 × 10^−19^
hsa00980:Metabolism of xenobiotics by cytochrome P450	1.83 × 10^−15^	3.60 × 10^−13^
hsa04810:Regulation of actin cytoskeleton	2.91 × 10^−14^	5.56 × 10^−12^
hsa04062:Chemokine signaling pathway	2.45 × 10^−11^	4.67 × 10^−09^
hsa04110:Cell cycle	7.71 × 10^−11^	1.47 × 10^−08^
hsa05220:Chronic myeloid leukemia	2.52 × 10^−10^	4.82 × 10^−08^
hsa04664:Fc epsilon RI signaling pathway	1.44 × 10^−09^	2.75 × 10^−07^
hsa03320:PPAR signaling pathway	2.73 × 10^−09^	5.22 × 10^−07^
hsa04910:Insulin signaling pathway	4.58 × 10^−09^	8.74 × 10^−07^
hsa04370:VEGF signaling pathway	4.63 × 10^−09^	8.85 × 10^−07^
hsa05215:Prostate cancer	7.36 × 10^−09^	1.41 × 10^−06^
hsa05221:Acute myeloid leukemia	2.93 × 10^−08^	5.60 × 10^−06^
hsa04270:Vascular smooth muscle contraction	1.07 × 10^−07^	2.05 × 10^−05^
hsa04670:Leukocyte transendothelial migration	1.17 × 10^−07^	2.24 × 10^−05^
hsa05218:Melanoma	1.30 × 10^−07^	2.48 × 10^−05^
hsa04722:Neurotrophin signaling pathway	3.39 × 10^−07^	6.47 × 10^−05^
hsa04520:Adherens junction	5.97 × 10^−07^	1.14 × 10^−04^
hsa05213:Endometrial cancer	1.32 × 10^−06^	2.51 × 10^−04^
hsa05214:Glioma	1.67 × 10^−06^	3.19 × 10^−04^
hsa04912:GnRH signaling pathway	2.80 × 10^−06^	5.34 × 10^−04^

Unlike conventional single-gene analysis of expression, the proposed discriminative module can include genes that do not individually pass the stringent criterion of the significance test, but are crucial when working collaboratively. Numerous metastasis-related genes, such as CAM genes (integrin α/β family), SMAD-family genes and genes encoding phosphoinositide-3-kinases (*PIK3CB*, *PIK3CG* and *PIK3R5*), were not significant in single-gene analysis, but were required for connecting other critical responsive genes. These metastasis-related genes might produce small effects that are steadily accumulated and amplified along the pathway; hence, only a module-based method can detect these genes.

### Inference of genetic architectures underlying the discriminative modules

**Figure 2.**
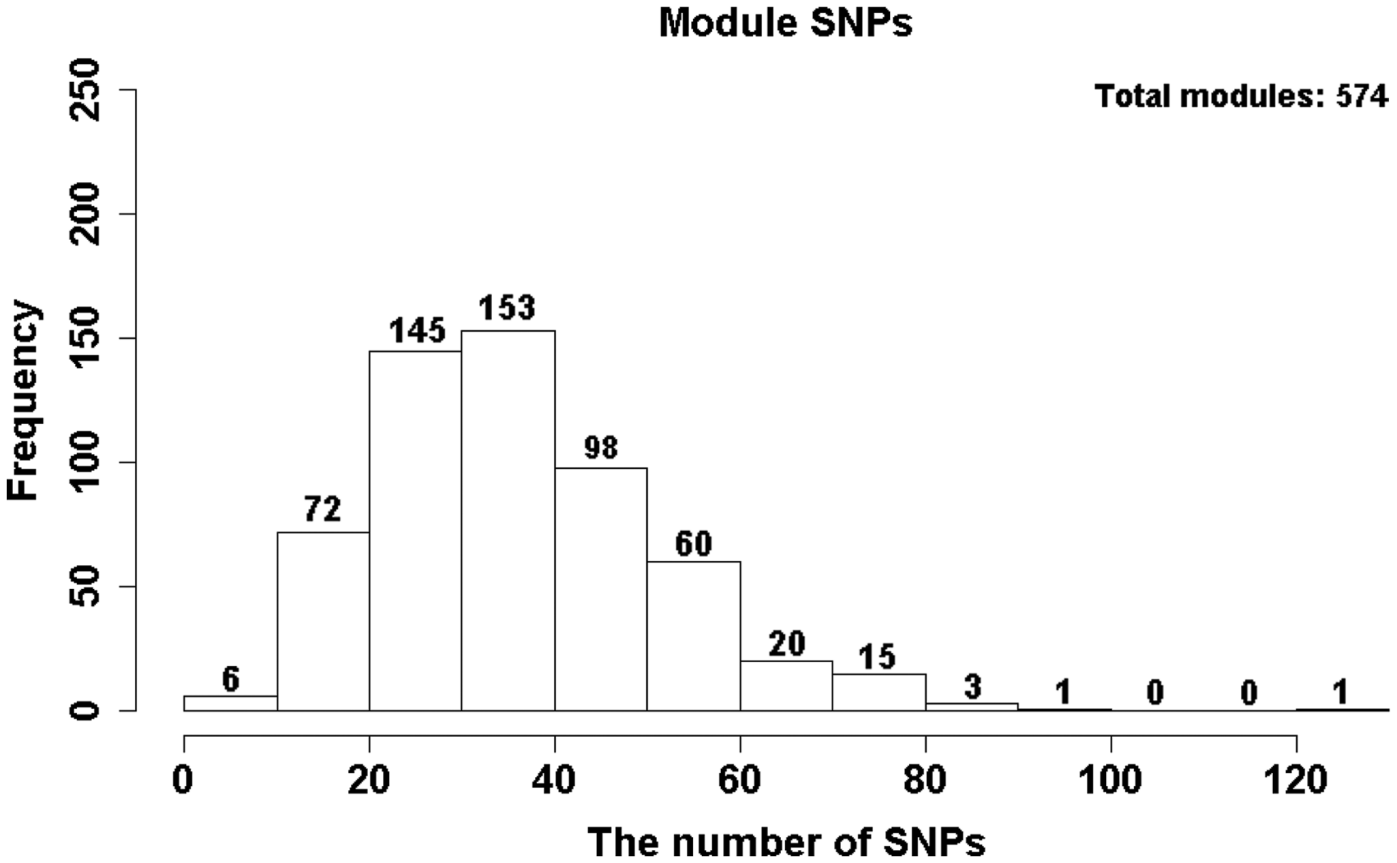
The distribution of the number of causal SNPs underlying the discriminative modules. Module-level eQTL mapping was performed with the SPLS method. A module of transcripts is considered as a multivariate response and is jointly analyzed by using multiple SNPs typed within 1 Mb of the transcriptional start or stop site of all module members.

**Figure 3.**
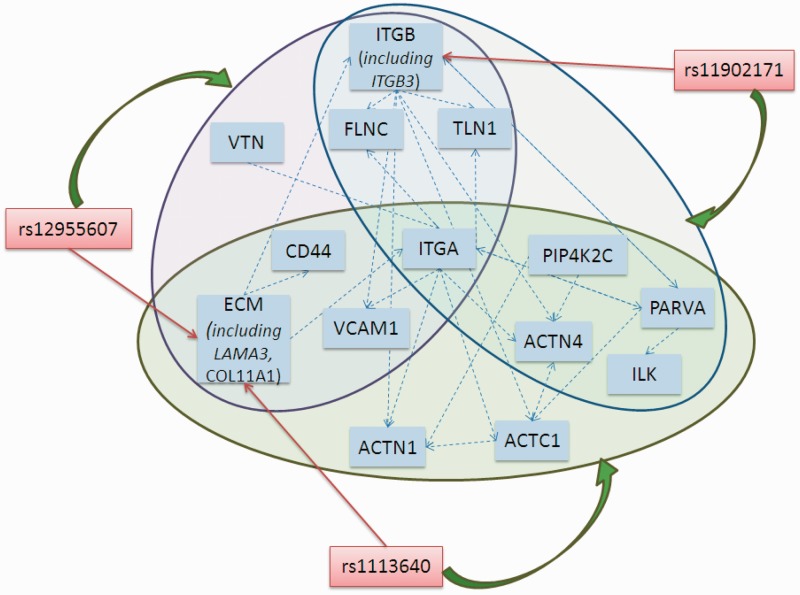
Focal adhesion linkage network. The circles enclose genes of the same module. The dotted lines are causal inferences that connect genes (blue squares) in the focal adhesion signaling pathway. Green arrows connect eQTLs (red squares) with significantly associated modules. The red solid edges connect eQTLs and their nearby genes. The locus rs11902171 physically links to *ITGB3* and rs12955607 physically links to *LAMA3*. Both of these loci are related to lymph node metastasis in OSCC. The results also show that rs1113640, which physically links to the CAM encoded by *COL11A1*, is another eQTL hotspot underlying focal adhesion signaling.

The next step was to identify the genetic variants that can explain the variations in module expression. We used SPSL regression to map eQTLs at the module level to identify multiple SNPs associated with multiple related genes in a discriminative module. Figure 2 illustrates the distribution of the number of eQTLs for the 574 discriminative modules; an average of 36 eQTLs was identified per module. The SNPs linked physically to genes encoding CAMs including laminins, collagens and integrins were most frequently selected as driving eQTLs and can thus be viewed as regulatory hotspots for module expression. Among these hotspots, the allelic markers that link to *LAMA3* and *ITGB3* have been shown to be susceptible to lymph node metastasis in OSCC (59,60). Both genes produce glycoproteins that mediate cell–ECM adhesion, cell migration, proliferation and survival. Furthermore, the SNP markers underlying the modules with most of the members belong to FAK signaling pathways are linked to CAMs (Figure 3). These eQTLs are probably the genetic basis of the variations observed in the expression of FAK-related modules across OSCC patients with and without nodal metastasis. Thus, these eQTL hotspots suggest that the linked CAMs likely cause an aberrant control of FAK signaling pathways, thereby promoting the metastatic phenotype. Other eQTL hotspots in our results also link to genes that are associated with cancer metastasis. For example, the S100-family proteins are well known to function in metastatic tumors (61–63): the secretion of tumor necrosis factor, transforming growth factor beta and VEGF-A from primary tumors upregulates the S100 chemokines, which in turn support the adhesion and dissemination of malignant cells in a calcium-dependent manner. The SNPs linked to the S100 chemokines may change the affinity of the chemokines to those inducing factors.

These observations indicate the potential paths through which DNA polymorphisms might affect nodal metastasis in OSCC. The SNPs associated with those selected modules are likely to be functionally relevant. Thus, leveraging causative eQTL information may facilitate the reconstruction of a causal gene network.

### Reconstruction of causal gene networks for lymph node metastasis

In this study, our primary goal was to reconstruct the causal gene network. A subnetwork was built with each pair of gene modules and their associated eQTLs using the Bayesian network approach (49). Moreover, the global causal gene network was reconstructed based on local causality inferences of multiple subnetworks to reduce the overall computational load. The challenge of this approach is that it may alter local causalities in the subnetwork in the context of the global gene network. To overcome this difficulty, we developed our framework to assemble the subnetwork based on a social ranking strategy proposed by Fushing *et al.* (38), and we named it random-field ranking in this study.

As mentioned in the preceding subsection, the metastasis-related focal adhesion signaling map was the most enriched pathway for the discriminative modules at a significance level of 0.05; most of the modules clustered in the actin-regulation branch and the phosphatidylinositol-signaling branch of the focal adhesion pathway. Studies have shown that the focal adhesion complex is involved in lymph node metastasis of head and neck cancer (64,65). This pathway is probably one of the key mechanisms mediating lymph node metastasis in OSCC. Thus, to test the proposed method, we demonstrated the causal network inference with the focal adhesion signaling pathway, whose causal relationships are mostly known in the KEGG database. Accordingly, this approach makes it possible to evaluate the consistency of the presented method with the experimentally verified connections. We selected 20 discriminative modules containing at least 2 genes on the 2 major chains of the signal cascade in the focal adhesion map involved in actin regulation and phosphatidylinositol signaling. Using these 20 discriminative modules, which are involved with the 147 genes and the 241 corresponding eQTLs, we attempted to reconstruct the main frame of the pathway.

**Figure 4.**
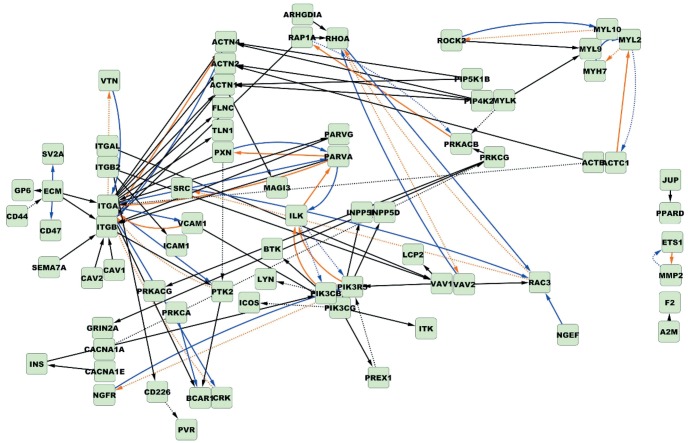
The combined focal adhesion pathway inferred based on the major direction and by using the random-field ranking strategy. Based on the 20 modules and their driving eQTLs, a focal adhesion network was reconstructed. The black edges were commonly identified based on the major direction decision and by using random-field ranking. The orange edges were predicted only by the major direction. The blue edges were constructed only in the random-field ranking procedure. Compared with the KEGG map, the solid and the dotted edges denote consistent and inverted orientations, respectively. Based on the major direction decision, the concordance rate was 77.21%. When multiple local causality networks were assembled by the random-field ranking procedure and the edge was oriented based on the global relative ranks of the corresponding pair of genes, the concordance rate was 83%.

As described in ‘Materials and Methods’ section, the directionality of a pair of genes can be evaluated more than once in multiple subnetworks and the edge orientation can be intuitively decided based on the major direction of the corresponding pair of genes (black and orange edges in Figure 4). Three gene families were overly represented in the map, and genes belonging to the same family mostly function in the same direction and on the same targets. Hence, these genes were combined into the same node (Figure 4). The three gene families include genes encoding ECM macromolecules and α/β integrins. The relative counts in the causation matrix were combined to infer the major directions. As Figure 4 shows, the focal adhesion pathway inferred by the major direction consists of 70 genes and 79 causal relationships that overlap the KEGG records. Among the 79 network edges, the causal inferences of 61 edges agree with the KEGG map, whereas the remaining 18 edges are inverted. The concordance rate is 77.21%. Because this study was focused on the causality inference, it does not consider the links documented in the KEGG database but not in our prediction. This is because the links in the KEGG database are comprehensive records, whereas certain regulations only function under specific conditions. We did not consider the links that were present in our inference but were not included in the KEGG collection because some of our inferences matched indirect links in KEGG and some inferences reflected potential novel regulations about which no concrete information was available. The results presented below indicate that the total number of links detected does not lead to potential bias of causality inference.

Performing the random-field ranking procedure, which was motivated by social network inference, generated the focal adhesion pathway that is denoted as black and blue edges in Figure 4. The edge orientation was determined using the random-field ranking coordinates of both genes. Of these edges, 69 edges were consistent with the KEGG map and the directions of 14 edges were inverted. The concordance rate is 83%, which is higher than the orientation deduced from major directions. The improvement over the simple decision of major directionality stems from the use information on indirect connections among common interplay partners, especially when neither of the two regulation directions inferred for a pair of genes dominates the other. Consequently, the edge orientations that cannot be restored based on the decision of major direction may be recovered by random-field ranking. For example, consider the link between BCAR1 and PRKCA. BCAR1 is a scaffolding and adaptor protein that functions in converging signals and determining cellular responses (66). BCAR1 becomes tyrosine phosphorylated in response to integrin engagement and activation of PRKCA, a family of serine- and threonine-specific protein kinases (67,68). The direction of the phosphorylation link from PRKCA to BCAR1 cannot be determined based on the major direction but can be recovered using the proposed random-field ranking strategy. Phosphorylation of BCAR1 in the focal adhesion pathway may contribute to cell adhesion, migration, oncogenic transformation and metastasis. This is because phosphorylated BCAR1 provides binding sites for the adaptor protein CRK, and the BCAR1/CRK complex induces cytoskeletal remodeling and promotes cell migration (69). Moreover, among the 18 inverted edges inferred based on the major direction, nine edges were corrected when random-field ranking was adopted (Figure 4). Some of the reversed instances that are known to play a part in amendatory integrin signaling flow are the following. Integrins are cell surface receptors that drive the focal adhesion pathway. The α-family integrins, *ITGA*, recognize the sequence R-G-D in a wide array of ligands including VTN, as corrected in Figure 4 (70,71). The α-family integrins also stimulate the downstream tyrosine kinase PTK2 and trigger BCAR1/CRK complex formation (72,73). The causal directions of this family were inferred conversely based on the major direction decision (dotted orange lines in Figure 4) and further corrected using the random-field ranking strategy (solid blue lines in Figure 4). The edges from *VAV2* to *RHOA* and from *ROCK2* to *MYL10* are two similar examples. *VAV2* encodes a guanine nucleotide exchange factor that catalyzes GDP/GTP exchange on RHOA GTPase. The ROCK2 kinase phosphorylates the myosin light chain encoded by *MYL10* to organize actin in the focal adhesion pathway. The nine edges corrected using random-field ranking (Figure 4) are primarily involved in cell adhesion and actin organization, which induce spreading and migration in tumor metastasis.

**Figure 5.**
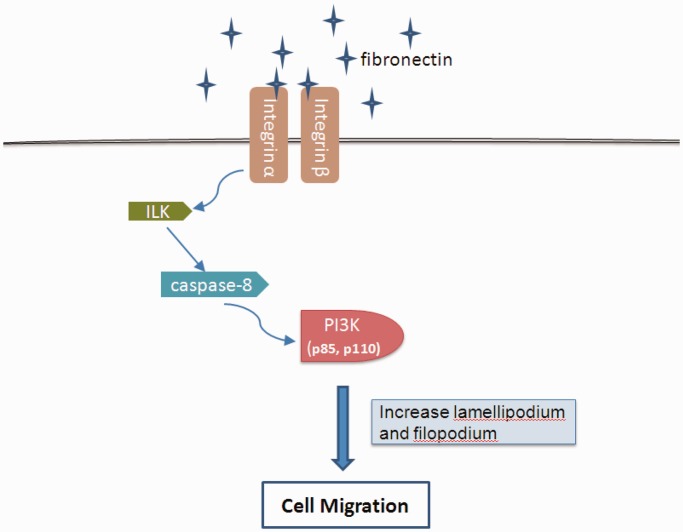
Cell migration is a critical process in the tumor invasion-metastasis cascade. Phosphatidylinositol 3-kinase (PI3K) is an important component of the cell migration apparatus. When integrin is activated through fibronectin binding, integrin-linked kinase (ILK) can activate Caspase-8. Caspase-8 interacts with the p85 regulatory subunit of PI3K, and p85 further binds to the catalytic subunit p110 (*PIK3CB*) of PI3K, thereby promoting cell migration.

The remaining reversed links could be either modeling errors, the presence of unobserved deregulated signals or alternative regulated pathways in OSCC nodal metastasis because gene activities are highly dynamic under various conditions. Among the 14 inverted relations (blue dotted lines in Figure 4), 12 were supported by previous studies (Supplementary Table S1), and some of these are the result of feedback loops or alternative pathways that have been documented in the literature but not curated in the KEGG database or in our molecular interaction data. For example, the edge from ILK to PI3K kinase is inverted likely because ILK activates Caspase-8 in an adhesion-dependent manner, and Caspase-8 further activates PI3K kinase to regulate cell adhesion and motility (Figure 5). Based on the high concordance rate obtained in this study, we expect there to be interesting novel relations worth exploring further.

The proposed framework attempts to identify representative eQTLs that regulate the expression patterns of discriminative modules. Although early studies indicated that including eQTLs was advantageous for network analysis, network constructions with and without eQTLs have not been compared on a large scale. To examine whether the information from eQTL mapping can facilitate causal relationship inference, we reconstructed the focal adhesion network following the same strategy described above but using only expression data. The concordance rate was only 56.75%, as expected for this type of analysis. More reversed associations occurred than before without the aid of SNPs to anchor the orientation. This result suggests that the driving eQTLs are critical determinants and effective anchors for orienting causal relationships (Supplementary Table S2).

To further evaluate the performance of our novel framework, we compared it with several existing alternative methods of causal network inference whose packages were available, including NEO (2), PC algorithm (74), QDG (5) and QTLnet (4). We tested these methods in the reconstruction of the focal adhesion signaling pathway with the 147 genes identified in the oral cancer data. With QDG and PC algorithm, multiple eQTLs are assumed to have been determined previously. With NEO, eQTL can be manually assigned or automatically selected before network edge orientation. For objective comparison, we input the same set of eQTLs identified using SPLS regression in all methods except for QTLnet; this is because, with the QTLnet method, the associated eQTL and causal network must be inferred simultaneously. As described earlier, we measured the percentage of network edges that were concordant with the experimentally confirmed pathways in the KEGG database. Table 3 lists the concordance rate of these methods. The QTLnet does not scale well >20 genes because the MCMC approach is used in this method. As to NEO, it yielded a concordance rate of 0.701, which compares favorably with those of the QDG and PC algorithm methods. Using the cancer eQTL data, we demonstrated that our method based on subnetwork assembly yielded a higher concordance rate (0.83) than the other algorithms tested. The network topology inferred by the proposed method is thus more consistent with the validated cancer pathways.

**Table 3.** Comparison of the methods for the causal network inference

	Subnetwork assembly		NEO	PC algorithm	QDG	QTLnet
Concordance rate		0.83	0.70	0.62	0.66	NA^a^

^a^The QTLnet cannot work on the number of nodes in the cancer data.

### Clinical application of the causal gene network in lymph node metastasis

**Figure 6.**
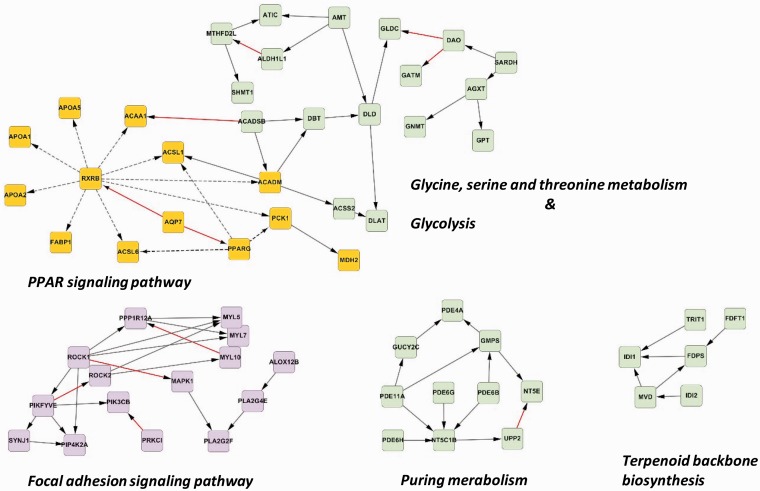
The causal gene network inferred from top discriminative modules at a significance level of 0.005. The major parts of the causal network that overlap with the KEGG maps are indicated. The network with green nodes involves metabolic pathways. The network with pink nodes provides a partial interface of the FAK signaling pathway, which communicates with the regulation of actin cytoskeleton and the MAPK signaling pathway. The network with yellow nodes mostly connected by dotted lines partially overlaps with the PPAR singling pathway, and the network is centered on *RXRB*. This is the first study to suggest that the *RXRB*-causal network is involved in lymph node metastasis.

The proposed method can reconstruct most parts of the most-enriched pathway (i.e. focal adhesion signaling). This network representation describes the information flow of the cellular process and can be used to develop novel hypotheses related to the regulatory mechanisms governing the phenotype of interest. Thus, to identify other pathways that are also activated, we reconstructed the causal network based on the 19 modules that were identified as most significant for distinguishing lymph node metastatic status by applying a stringent *P* < 0.005. Our results indicate that several parts of the causal network overlap with well-known pathways in the KEGG database (Figure 6). The novel RXRB-causal network (yellow in Figure 6) exhibited a unique phenomenon: the network, which partially appeared on the KEGG PPAR signaling map, could only be reconstructed using data of patients diagnosed as node-negative, but the network collapsed when data of node-positive patients alone were used. We postulate that the RXRB-causal network might be highly dysregulated under the condition of nodal metastasis and thus no longer coordinated effectively. Accordingly, we next tested whether the newly identified RXRB-causal network could be a novel signature associated with OSCC nodal status. Among the 13 genes in the inferred RXRB-causal network, stepwise logistic regression identified a six-gene model (*MDH2*, *RXRB*, *FABP1*, *ACADM*, *APOA5* and *PPARG*) as the most predictive model of lymph node metastasis. The performance of the six-gene model was assessed using the receiver operator characteristic, which showed an area under the curve of 0.925 (Supplementary Figure S2). Thus, this molecular signature is potentially a novel indicator of nodal metastasis in OSCC.

We also investigated the effects of the *RXRB*-causal network on clinical outcomes by using multivariate Cox regression analysis. The results indicate that 8 out of the 13 genes in this network are significantly associated with increased risks of neck relapse, distant metastasis and poor disease-free, disease-specific and overall survival (Table 4). The aberrant *RXRB*-causal network not only occurs in patients with nodal metastasis, but also leads to a poor prognosis in OSCC. The expression level of the hub gene *RXRB* is significantly associated with all clinical outcomes listed, and its hazard ratios are relatively high. This is because mutations of hub genes have been demonstrated to alter the network dynamics of cancer cells (24). Thus, *RXRB* is likely to play a role in organizing the communication and functions of this causal network. Consequently, the downstream effectors of *RXRB* also exhibit detrimental effects on tumor relapse and poor survival in OSCC.

**Table 4.** Cox regression analysis of different time-to-event clinical traits with the RXRB-causal network

	Neck relapse		Distant metastases		Disease-free survival		Disease-specific survival		Overall survival	
	Hazard ratio	*P*	Hazard ratio	*P*	Hazard ratio	*P*	Hazard ratio	*P*	Hazard ratio	*P*
MDH2			7.328	0.039						
RXRB	16.539	0.027	61.028	0.005	20.007	0.003	24.037	0.003	14.978	0.005
APOA1	0.037	0.001	0.016	0.001	0.12	0.023	*0.022*	*0.00004*	0.029	0.000025
AQP7	12.147	0.003	35.424	0.003	12.183	0.000175	6.654	0.004	10.185	0.000186
ACADM	0.289	0.026			0.319	0.01	0.369	0.053	0.411	0.053
ACSL1			0.187	0.002			0.463	0.04	0.436	0.013
APOA5					0.143	0.059				
ACSL6									0.217	0.058

## DISCUSSION

In this study, we developed a four-step method with reduced computational load to reconstruct causal gene network for a complex phenotype. Because of the high-dimensional nature of genomic data, we preselected a moderate number of phenotypically motivated variables using phenotype-specific modules and causal eQTLs. Our hybrid scoring function is more desirable than conventional module identification because genes in a molecular interactome have previously been suggested to be of two types (24,75). Genes of one type encode static protein complexes, in which gene products interact with each other concurrently. Thus, these genes are overexpressed or downregulated altogether under distinct conditions and thus exhibit highly correlated coexpression patterns across diverse cellular states. Genes of the second type act as intermediate communicators that convey signals between distinct protein complexes or pathways under distinct conditions. Thus, genes of this type interact with distinct partners under dissimilar conditions and display condition-specific correlation structures. Therefore, the hybrid scoring method considers not only the general expression correlation for a pair of interacting molecules, but also the changes in their coexpression patterns between conditions. Based on the human interactome, the module-based strategy can identify genes that are differentially expressed with less-significant *P* values and are essential for forming a protein complex or organizing the interconnectivity of other responsive genes. The integrins in the focal adhesion pathway and *PPARG* in the PPAR signaling pathway are two such examples.

The eQTL mapping process provides a causal experimental system in which genotype drives expression variations of the phenotype-associated modules. Recently, regulatory networks have been built by integrating eQTLs. An inherent problem with the increasing number of nodes is the exponential growth of the search space. The results of this study demonstrate that constructing multiple small local subnetworks in parallel is an efficient and effective method for assembling a full-scale causal network. In this novel framework, the local subnetwork inference is performed on each pairwise module rather than on individual modules. In this manner, the directionality of most gene pairs can be evaluated a sufficient number of times in the context of distinct combinations of modules. The repeated testing of the causal relationship between two genes in the context of distinct neighborhoods can also avoid spurious association because random association will not be repeated at a high rate.

This is the first study to apply the concept of dominance ranking from animal social network analysis to assemble multiple local gene networks. The inference of global causal interrelationships from local causality tests and the deduction of transitive causalities between any two genes connected by a path are central to this process. The main advantage of the proposed framework is that it decreases computational complexity by excluding phenotype-irrelevant variables and avoiding a search over the entire network. Our results also indicate that integrating eQTLs hold further promise for inferring causal relationships.

The phenotype-associated causal gene network provides a potential basis for understanding the driving mechanisms of complex diseases and identifying new targets to combat those diseases. In this study, we used the proposed multistep procedure to examine lymph node metastasis in OSCC. Applying the proposed method, a major part of the focal adhesion signaling network was reconstructed using tumor samples. The result indicates that most of the causal relationships within a pathway were retained in the cancer tissue. The inconsistent edges in the network probably arise at least partly from disturbed information flows, rather than just from modeling errors. The disturbed information flow may imply an unexpected complexity generated in the causal network by certain unobserved signals; however, it may also implicate novel alternative modification pathways that affect network connectivity and directionality, such as DNA methylation or protein modification. This study provides a foundation for future investigations on disturbed information flows in cancer progression, growth and metastasis.

The findings presented herein reveal a novel gene network, the *RXRB*-causal network, for lymph node metastasis in OSCC. The dynamic structure of this causal network was disorganized in the node-positive group, and thus the network could not be reconstructed using the data of node-positive patients alone. Conversely, the network integrity and interconnectivity were maintained in the node-negative group. In this network, *RXRB* is upstream of almost all the remaining genes. This topology suggests a driving role of *RXRB* in regulating other transcripts. *RXRB* encodes a type of nuclear receptor that is a ligand-activated transcription factor. The RXRB protein can activate transcription by functioning as a homodimer or serve as a heterodimerization partner for other Type 2 nuclear receptors, such as the peroxisome proliferator-activated receptors (PPARG) in the *RXRB*-causal network. Thus, RXRB functions as a causal regulator of multiple signaling pathways that range from cell proliferation to lipid metabolism (76). The well-known process of retinoid binding to RXRB leads to the regulation of cell growth, differentiation, apoptosis and proliferation (77). RXRB immunoreactivity and abnormal expression of RXRB mRNA have been disclosed to be associated with the status of lymph node metastasis in esophageal squamous cell carcinoma (78), and they may also be related to other carcinoma progressions (76). We speculate that the inferred *RXRB*-causal network mediates, at least in part, the metastasis to cervical lymph nodes in OSCC. The information flows in the network may have been highly perturbed in the nodal-positive samples. Thus, the modulization of the *RXRB*-causal network may have been destroyed in the node-positive group because of a loss of coordinated expression patterns. Accordingly, the *RXRB*-causal network likely plays critical roles in inhibiting lymph node spreading in OSCC. To the best of our knowledge, there are no reports on the clinical significance of the *RXRB*-related network in OSCC. However, the results of this study demonstrate the ability of a six-gene model to predict OSCC nodal metastasis. Clinicopathological factors, such as tumor size and location, are not strongly predictive of lymph node metastasis in intermediate-risk OSCC, and ∼70% of clinically node-negative patients undergo unnecessary surgical resection of the neck lymph nodes. An ongoing challenge is to reduce unnecessary neck surgery in clinically node-negative patients. Because little is known about the genetic biomarkers of occult metastasis, the genes in the *RXRB*-causal network can potentially be used to detect occult nodal metastasis among clinically node-negative patients. Thus, further investigating the prediction model in larger studies might improve current clinical decision-making.

The results of this study also demonstrate that expression changes in the genes of the inferred *RXRB*-causal network are significantly associated with increased risks of tumor relapse, distant metastases and poor survival outcomes. Among these genes, the key driver, *RXRB*, exhibits the strongest effect on poor prognosis. This novel molecular profiling may potentially change treatment modalities or elicit a shift from conservative to more aggressive approaches for treating high-risk patients. Our findings suggest that the detailed functions of the *RXRB*-causal network in tumor metastasis should be investigated further. Identifying selective synthetic ligands for the RXRB driver might lead to the development of a novel therapeutic control of dysregulated pathways in OSCC.

In conclusion, casual gene network inference is an effective approach for understanding complex disease phenotypes and identifying potential key drivers. Changes in the network modularity could be a defining feature for distinct disease statuses. Conventional hypothesis testing is time-consuming and is limited to a few causal relationships, whereas the proposed method facilitates the generation of new hypotheses based on genomic eQTLs and expression data. An in-depth clarification of the causal interrelationships not only helps prioritize the potential hypotheses, but also aids in the development of novel therapeutic strategies. Measuring the changes in a causal network is likely to offer profound insight into the dysfunctional regulatory systems and improve the predictive value of traditional clinical indicators.

## SUPPLEMENTARY DATA

Supplementary Data are available at NAR Online.

## FUNDING

Funding for open access charge: The National Council of Science [NSC101-2325-B-007-003 and 99-2628-B-182A-002-MY3].

*Conflict of interest statement*. None declared.

## Supplementary Material

Supplementary Data

## References

[1] Schadt EE, Lamb J, Yang X, Zhu J, Edwards S, Guhathakurta D, Sieberts SK, Monks S, Reitman M, Zhang C (2005). An integrative genomics approach to infer causal associations between gene expression and disease. Nat. Genet..

[2] Aten JE, Fuller TF, Lusis AJ, Horvath S (2008). Using genetic markers to orient the edges in quantitative trait networks: the NEO software. BMC Syst. Biol..

[3] Zhu J, Wiener MC, Zhang C, Fridman A, Minch E, Lum PY, Sachs JR, Schadt EE (2007). Increasing the power to detect causal associations by combining genotypic and expression data in segregating populations. PLoS Comput. Biol..

[4] Neto EC, Keller MP, Attie AD, Yandell BS (2010). Causal graphical models in systems genetics: a unified framework for joint inference of causal network and genetic architecture for correlated phenotypes. Ann. Appl. Stat..

[5] Chaibub Neto E, Ferrara CT, Attie AD, Yandell BS (2008). Inferring causal phenotype networks from segregating populations. Genetics.

[6] Liu B, de la Fuente A, Hoeschele I (2008). Gene network inference via structural equation modeling in genetical genomics experiments. Genetics.

[7] Li R, Tsaih SW, Shockley K, Stylianou IM, Wergedal J, Paigen B, Churchill GA (2006). Structural model analysis of multiple quantitative traits. PLoS Genet..

[8] Valente BD, Rosa GJ, de Los Campos G, Gianola D, Silva MA (2010). Searching for recursive causal structures in multivariate quantitative genetics mixed models. Genetics.

[9] Winrow CJ, Williams DL, Kasarskis A, Millstein J, Laposky AD, Yang HS, Mrazek K, Zhou L, Owens JR, Radzicki D (2009). Uncovering the genetic landscape for multiple sleep-wake traits. PloS One.

[10] Logsdon BA, Mezey J (2010). Gene expression network reconstruction by convex feature selection when incorporating genetic perturbations. PLoS Comput. Biol..

[11] Hageman RS, Leduc MS, Korstanje R, Paigen B, Churchill GA (2011). A Bayesian framework for inference of the genotype-phenotype map for segregating populations. Genetics.

[12] Horvath S, Zhang B, Carlson M, Lu KV, Zhu S, Felciano RM, Laurance MF, Zhao W, Qi S, Chen Z (2006). Analysis of oncogenic signaling networks in glioblastoma identifies ASPM as a molecular target. Proc. Natl Acad. Sci. USA.

[13] Miller JA, Oldham MC, Geschwind DH (2008). A systems level analysis of transcriptional changes in Alzheimer's disease and normal aging. J. Neurosci..

[14] Keller MP, Choi Y, Wang P, Davis DB, Rabaglia ME, Oler AT, Stapleton DS, Argmann C, Schueler KL, Edwards S (2008). A gene expression network model of type 2 diabetes links cell cycle regulation in islets with diabetes susceptibility. Genome Res..

[15] Presson AP, Sobel EM, Papp JC, Suarez CJ, Whistler T, Rajeevan MS, Vernon SD, Horvath S (2008). Integrated weighted gene co-expression network analysis with an application to chronic fatigue syndrome. BMC Syst. Biol..

[16] Langfelder P, Horvath S (2008). WGCNA: an R package for weighted correlation network analysis. BMC Bioinformatics.

[17] Gu J, Chen Y, Li S, Li Y (2010). Identification of responsive gene modules by network-based gene clustering and extending: application to inflammation and angiogenesis. BMC Syst. Biol..

[18] Chuang HY, Lee E, Liu YT, Lee D, Ideker T (2007). Network-based classification of breast cancer metastasis. Mol. Syst. Biol..

[19] Hwang T, Park T (2009). Identification of differentially expressed subnetworks based on multivariate ANOVA. BMC Bioinformatics.

[20] Ideker T, Ozier O, Schwikowski B, Siegel AF (2002). Discovering regulatory and signalling circuits in molecular interaction networks. Bioinformatics.

[21] Guo Z, Wang L, Li Y, Gong X, Yao C, Ma W, Wang D, Zhu J, Zhang M, Yang D (2007). Edge-based scoring and searching method for identifying condition-responsive protein-protein interaction sub-network. Bioinformatics.

[22] Cabusora L, Sutton E, Fulmer A, Forst CV (2005). Differential network expression during drug and stress response. Bioinformatics.

[23] Sun Z, Luo J, Zhou Y, Liu K, Li W (2009). Exploring phenotype-associated modules in an oral cavity tumor using an integrated framework. Bioinformatics.

[24] Taylor IW, Linding R, Warde-Farley D, Liu Y, Pesquita C, Faria D, Bull S, Pawson T, Morris Q, Wrana JL (2009). Dynamic modularity in protein interaction networks predicts breast cancer outcome. Nat. Biotechnol..

[25] Zhang X, Yang H, Gong B, Jiang C, Yang L (2012). Combined gene expression and protein interaction analysis of dynamic modularity in glioma prognosis. J. Neurooncol..

[26] Lander ES, Botstein D (1989). Mapping mendelian factors underlying quantitative traits using RFLP linkage maps. Genetics.

[27] Kao CH, Zeng ZB, Teasdale RD (1999). Multiple interval mapping for quantitative trait loci. Genetics.

[28] Zou W, Zeng ZB (2009). Multiple interval mapping for gene expression QTL analysis. Genetica.

[29] Wang P, Dawson JA, Keller MP, Yandell BS, Thornberry NA, Zhang BB, Wang IM, Schadt EE, Attie AD, Kendziorski C (2011). A model selection approach for expression quantitative trait loci (eQTL) mapping. Genetics.

[30] Jia Z, Xu S (2007). Mapping quantitative trait loci for expression abundance. Genetics.

[31] Brem RB, Kruglyak L (2005). The landscape of genetic complexity across 5700 gene expression traits in yeast. Proc. Natl Acad. Sci. USA.

[32] Lee SI, Dudley AM, Drubin D, Silver PA, Krogan NJ, Pe'er D, Koller D (2009). Learning a prior on regulatory potential from eQTL data. PLoS Genet..

[33] Chun H, Keles S (2009). Expression quantitative trait loci mapping with multivariate sparse partial least squares regression. Genetics.

[34] Chun H, Keles S (2010). Sparse partial least squares regression for simultaneous dimension reduction and variable selection. J. R. Stat. Soc. Series B Stat. Methodol..

[35] Chase ID, Seitz K (2011). Self-structuring properties of dominance hierarchies a new perspective. Adv. Genet..

[36] de VH (1998). Finding a dominance order most consistent with a linear hierarchy: a new procedure and review. Anim. Behav..

[37] Jameson KA, Appleby MC, Freeman LC (1999). Finding an appropriate order for a hierarchy based on probabilistic dominance. Anim. Behav..

[38] Fushing H, McAssey MP, Beisner B, McCowan B (2011). Ranking network of a captive rhesus macaque society: a sophisticated corporative kingdom. PloS One.

[39] Adams ES (2005). Bayesian analysis of linear dominance hierarchies. Anim. Behav..

[40] Stark C, Breitkreutz BJ, Chatr-Aryamontri A, Boucher L, Oughtred R, Livstone MS, Nixon J, Van Auken K, Wang X, Shi X (2011). The BioGRID Interaction Database: 2011 update. Nucleic Acids Res..

[41] Kanehisa M, Goto S, Sato Y, Furumichi M, Tanabe M (2012). KEGG for integration and interpretation of large-scale molecular data sets. Nucleic Acids Res..

[42] Peng CH, Liao CT, Peng SC, Chen YJ, Cheng AJ, Juang JL, Tsai CY, Chen TC, Chuang YJ, Tang CY (2011). A novel molecular signature identified by systems genetics approach predicts prognosis in oral squamous cell carcinoma. PloS One.

[43] Hsu JT, Peng CH, Hsieh WP, Lan CY, Tang CY (2011). A novel method to identify cooperative functional modules: study of module coordination in the *Saccharomyces cerevisiae* cell cycle. BMC Bioinformatics.

[44] Heyer LJ, Kruglyak S, Yooseph S (1999). Exploring expression data: identification and analysis of coexpressed genes. Genome Res..

[45] Hastie T, Zou H (2005). Regularization and variable selection via the elastic net. J. R. Stat. Soc..

[46] Frank I, Friedman J (1993). A statistical view of some chemometrics regression tools. Technometrics.

[47] Morley M, Molony CM, Weber TM, Devlin JL, Ewens KG, Spielman RS, Cheung VG (2004). Genetic analysis of genome-wide variation in human gene expression. Nature.

[48] Wang K, Li M, Bucan M (2007). Pathway-based approaches for analysis of genomewide association studies. Am. J. Hum. Genet..

[49] Susanne G, Boettcher CD (2003). Deal: a package for learning bayesian network. J. Stat. Softw..

[50] Huang da W, Sherman BT, Tan Q, Kir J, Liu D, Bryant D, Guo Y, Stephens R, Baseler MW, Lane HC (2007). DAVID Bioinformatics Resources: expanded annotation database and novel algorithms to better extract biology from large gene lists. Nucleic Acids Res..

[51] Huang da W, Sherman BT, Lempicki RA (2009). Systematic and integrative analysis of large gene lists using DAVID bioinformatics resources. Nat. Protoc..

[52] Ashburner M, Ball CA, Blake JA, Botstein D, Butler H, Cherry JM, Davis AP, Dolinski K, Dwight SS, Eppig JT (2000). Gene ontology: tool for the unification of biology. The Gene Ontology Consortium. Nat. Genet..

[53] Jiang JG, Ning YG, Chen C, Ma D, Liu ZJ, Yang S, Zhou J, Xiao X, Zhang XA, Edin ML (2007). Cytochrome p450 epoxygenase promotes human cancer metastasis. Cancer Res..

[54] Yu W, Chen L, Yang YQ, Falck JR, Guo AM, Li Y, Yang J (2011). Cytochrome P450 omega-hydroxylase promotes angiogenesis and metastasis by upregulation of VEGF and MMP-9 in non-small cell lung cancer. Cancer Chemother. Pharmacol..

[55] Pardali K, Moustakas A (2007). Actions of TGF-beta as tumor suppressor and pro-metastatic factor in human cancer. Biochim. Biophys. Acta.

[56] Sawai H, Okada Y, Funahashi H, Matsuo Y, Takahashi H, Takeyama H, Manabe T (2005). Activation of focal adhesion kinase enhances the adhesion and invasion of pancreatic cancer cells via extracellular signal-regulated kinase-1/2 signaling pathway activation. Mol. Cancer.

[57] Parsons JT, Slack-Davis J, Tilghman R, Roberts WG (2008). Focal adhesion kinase: targeting adhesion signaling pathways for therapeutic intervention. Clin. Cancer Res..

[58] Zhao J, Guan JL (2009). Signal transduction by focal adhesion kinase in cancer. Cancer Metastasis Rev..

[59] Snijders AM, Schmidt BL, Fridlyand J, Dekker N, Pinkel D, Jordan RC, Albertson DG (2005). Rare amplicons implicate frequent deregulation of cell fate specification pathways in oral squamous cell carcinoma. Oncogene.

[60] Ma XR, Cheng H, Wang XY, Liu H, Zhao D (2012). Single-nucleotide polymorphisms of integrins are associated with the risk and lymph node metastasis of oral squamous cell carcinoma. Med. Oncol..

[61] Ichikawa M, Williams R, Wang L, Vogl T, Srikrishna G (2011). S100A8/A9 activate key genes and pathways in colon tumor progression. Mol. Cancer Res..

[62] Rafii S, Lyden D (2006). S100 chemokines mediate bookmarking of premetastatic niches. Nat. Cell Biol..

[63] Sapkota D, Bruland O, Boe OE, Bakeer H, Elgindi OA, Vasstrand EN, Ibrahim SO (2008). Expression profile of the S100 gene family members in oral squamous cell carcinomas. J. Oral. Pathol. Med..

[64] Jiang H, Liu L, Ye J, Liu H, Xing S, Wu Y (2010). Focal adhesion kinase serves as a marker of cervical lymph node metastasis and is a potential therapeutic target in tongue cancer. J. Cancer Res. Clin. Oncol..

[65] Canel M, Secades P, Rodrigo JP, Cabanillas R, Herrero A, Suarez C, Chiara MD (2006). Overexpression of focal adhesion kinase in head and neck squamous cell carcinoma is independent of fak gene copy number. Clin. Cancer Res..

[66] Bouton AH, Riggins RB, Bruce-Staskal PJ (2001). Functions of the adapter protein Cas: signal convergence and the determination of cellular responses. Oncogene.

[67] Fagerstrom S, Pahlman S, Nanberg E (1998). Protein kinase C-dependent tyrosine phosphorylation of p130cas in differentiating neuroblastoma cells. J. Biol. Chem..

[68] Petch LA, Bockholt SM, Bouton A, Parsons JT, Burridge K (1995). Adhesion-induced tyrosine phosphorylation of the p130 src substrate. J. Cell Sci..

[69] Sakai R, Iwamatsu A, Hirano N, Ogawa S, Tanaka T, Mano H, Yazaki Y, Hirai H (1994). A novel signaling molecule, p130, forms stable complexes *in vivo* with v-Crk and v-Src in a tyrosine phosphorylation-dependent manner. EMBO J..

[70] Schnapp LM, Hatch N, Ramos DM, Klimanskaya IV, Sheppard D, Pytela R (1995). The human integrin alpha 8 beta 1 functions as a receptor for tenascin, fibronectin, and vitronectin. J. Biol. Chem..

[71] Nip J, Brodt P (1995). The role of the integrin vitronectin receptor, alpha v beta 3 in melanoma metastasis. Cancer Metastasis Rev..

[72] Mielenz D, Hapke S, Poschl E, von Der Mark H, von Der Mark K (2001). The integrin alpha 7 cytoplasmic domain regulates cell migration, lamellipodia formation, and p130CAS/Crk coupling. J. Biol. Chem..

[73] Zachary I, Rozengurt E (1992). Focal adhesion kinase (p125FAK): a point of convergence in the action of neuropeptides, integrins, and oncogenes. Cell.

[74] Li J, Wang Z, McKeown MJ (2008). Learning brain connectivity with the false-discovery-rate-controlled PC-algorithm. Conf. Proc. IEEE Eng. Med. Biol. Soc..

[75] Han JD, Bertin N, Hao T, Goldberg DS, Berriz GF, Zhang LV, Dupuy D, Walhout AJ, Cusick ME, Roth FP (2004). Evidence for dynamically organized modularity in the yeast protein-protein interaction network. Nature.

[76] Lefebvre P, Benomar Y, Staels B (2010). Retinoid X receptors: common heterodimerization partners with distinct functions. Trends Endocrinol. Metab..

[77] Wu K, Zhang Y, Xu XC, Hill J, Celestino J, Kim HT, Mohsin SK, Hilsenbeck SG, Lamph WW, Bissonette R (2002). The retinoid X receptor-selective retinoid, LGD1069, prevents the development of estrogen receptor-negative mammary tumors in transgenic mice. Cancer Res..

[78] Fujishima F, Suzuki T, Nakamura Y, Taniyama Y, Ono K, Sugawara A, Miyazaki S, Moriya T, Sato A, Satomi S (2011). Retinoid receptors in human esophageal squamous cell carcinoma: retinoid X receptor as a potent prognostic factor. Pathol. Int..

