# Supplementary material for: Causal inference of gene regulation with subnetwork assembly from genetical genomics data
Source: Nucleic Acids Res. 2013 Dec 9;42(5):2803–19. doi: 10.1093/nar/gkt1277 (PMC3950678; doi:10.1093/nar/gkt1277)
Supplement: Supplementary Data [file supp_42_5_2803__index.html]

Causal inference of gene regulation with subnetwork assembly from genetical genomics data — Causal inference of gene regulation with subnetwork assembly from genetical genomics data — Supplementary Data 

# Causal inference of gene regulation with subnetwork assembly from genetical genomics data

## Supplementary Data

files

**Files in this Data Supplement:**

- Supplementary Data - docx file
