# Supplementary material for: Causal inference of gene regulation with subnetwork assembly from genetical genomics data
Source: Nucleic Acids Res. 2013 Dec 9;42(5):2803–19. doi: 10.1093/nar/gkt1277 (PMC3950678; doi:10.1093/nar/gkt1277)

# Supplementary Methods

## Hotelling’s T-test for Identification of discriminative modules

Each potential new member joins current members to form Hotelling’s T-test for the expression difference between Disease States 1 and 2. The following null hypotheses are of interest

$$H_{0}:\boldsymbol{\mu}_{\mathbf{1}}=\boldsymbol{\mu}_{\mathbf{2}}$$

where $\boldsymbol{\mu}_{\mathbf{i}}$ is the expected value of gene expression with disease Status i for I = 1,2. The test statistic is as follows:

$$T^{2}=\left( {\bar{\mathbf{X}}}_{\mathbf{1}}-{\bar{\mathbf{X}}}_{\mathbf{2}} \right)^{'}\left[ \left( \frac{1}{n_{1}}+\frac{1}{n_{2}} \right)\mathbf{S}_{\mathrm{pooled}} \right]^{-1}\left( {\bar{\mathbf{X}}}_{\mathbf{1}}-{\bar{\mathbf{X}}}_{\mathbf{2}} \right)$$

where T^2^ is distributed as $\frac{\left( n_{1}+n_{2}-2 \right)k}{\left( n_{1}+n_{2}-k-1 \right)}F_{k,n_{1}+n_{2}-k-1}$ under the null hypothesis, and ${\bar{\mathbf{X}}}_{\mathbf{i}}$ is the sample mean of the gene expression with Disease Status i for i= 1,2 and $\mathbf{S}_{\mathrm{pooled}}=\frac{n_{1}-1}{n_{1}+n_{2}-2}\mathbf{S}_{1}+\frac{n_{2}-1}{n_{1}+n_{2}-2}\mathbf{S}_{2}$ , **S**_i_ is the sample covariance of the gene expression with Disease Status i for i= 1,2, $n_{i}$ is the number of patients with Disease Status i for i=1,2 , and k is the number of genes. If the neighbor with the highest bonding score passes a test with a significance level of 0.05, it is included in the module. Otherwise, the neighbor with the second highest bonding score joins the existing members for the test. If no tests are significant, the module stops expanding.

## The cost function in Random-field ranking procedure for local gene network assembly

For each of the *K* matrices of the enhanced control potential $P_{ij}^{*(k)}$ generated from beta distribution, we derive the optimal ranking coordinates for all genes following the method of Fushing et al*.* and employed simulated annealing to determine the optimal order:

$$\sigma^{(k)}=\mathop{\mathrm{argmin}}\limits_{\sigma} \sum_{i>j} H\left(\left\langle P_{ij}^{*(k)}\right\rangle_{\sigma}\right)G(i,j)$$

where $H(p)=\max(0,-2\log[2(1-p)])$ and $G(i,j)=\exp[(N+1-j)(i-j)/N^{2}]$. For each element in the lower triangle of $P^{*(k)}$, larger $P_{ij}^{*(k)}$ and larger difference in (i – j) is associated with greater total cost. Because the cost for the optimized mean field $\bar{P}_{K}^{*}=\sum_{k=1}^{K} P^{*(k)}$ was less than the cost for any optimized individual matrix $P^{*(k)}$, the optimal set $\bar{\sigma}^{(k)}$ of $\bar{P}_{K}^{*}$ was adopted as estimates of the relative ranks for the genes in the causation matrix.

# Significance of the potential factors for causal network reconstruction

Some parameters can potentially affect the results of the proposed causal network reconstruction method. These include the number of replications required to generate control probability matrix (K), number of intermediate neighbors used to infer transitive causal relations (R), and whether to merge the gene families in the causation matrix. To evaluate the contribution of each factor for causal inference, this study reconstructed the network for focal adhesion using various parameter combinations. A linear model was used to analyze the effect of each factor regarding the concordance rate of causal inferences compared to the regulation direction documented in the KEGG database. This model was constructed as follows:

$$y_{\mathrm{ijkp}}=\mu+\alpha_{i}+\beta_{j}+\gamma_{k}+\delta_{p}+\varepsilon_{\mathrm{ijkp}}$$

where $y_{\mathrm{ijkp}}$ is the concordance rate; $\alpha_{i},\beta_{j}, \gamma_{k}\mathrm{and}\delta_{p}$ are the main effects for R (=1 or 2), K(=100 or 1000), whether to include eQTL and whether to perform the merging process; and $\varepsilon_{\mathrm{ijkp}}$ is an error term that follows a normal distribution. The significance of the four factors is summarized in Supplementary Table S2. Merging gene families in the causation matrix had a negligible effect on the proposed framework. The concordance rates were insensitive to the parameters R, K. Only eQTL was a significant factor (*p*-value 0.00423).

This study set K = 1000 as the default, as in Fushing et al. (Fushing et al, 2011), and R = 1 for the transitive causalities of the common interacting neighbors between two genes. A merging process for large gene families was performed to illustrate constructed network in a feasible scheme similar to that of KEGG.

# References

Chun H, Keles S (2009) Expression quantitative trait loci mapping with multivariate sparse partial least squares regression. *Genetics* **182:** 79-90

Chun H, Keles S (2010) Sparse partial least squares regression for simultaneous dimension reduction and variable selection. *J R Stat Soc Series B Stat Methodol* **72:** 3-25

Fushing H, McAssey MP, Beisner B, McCowan B (2011) Ranking network of a captive rhesus macaque society: a sophisticated corporative kingdom. *PLoS One* **6:** e17817

Supplementary Table S1. The potential alternative paths for the 14 relations which are inconsistent with KEGG FAK signaling map

| Causal inference | Description | Reference |
| --- | --- | --- |
| ACTB to ITGA | ACTB transcriptionally activates SRC1. SRC1 further transcriptionally activates ITGA5 | [[1](#_ENREF_1)], [[2](#_ENREF_2)] |
| CACNA1A to PRKCG | CACNA1A, a calcium channel transports cytosolic Ca2+.  Cytosolic Ca2+ binds to PKC-gamma and activates it. | [3], [4], [5], [6], [7] |
| CD226 to PVR | N/A |  |
| CD44 to ECM | CD44 physically interacts with MMP-2 and increases its activity. Then, MMP2 inhibits LAMA2. | [8],[9] |
| ILK to PIK3R5 | ILK phosphorylates AKT1 and increases its activity. AKT1 further phosphorylates ESR1, thereby activating G-protein gamma. It finally activates PIK3R5 | [10], [11],[12], [13] |
| ILK to PIK3CB | ILK physically interacts with Caspase-8 and increases its activity. Caspase-8 then activates PI3K reg class IA (p85-alpha). Finally, Complex regulatory subunit PI3K reg class IA (p85) binds to catalytic subunit PIK3CB and activates it. | [14], [15],[16] |
| MMP2 to ETS1 | MMP-2 C cleaves GSK3B, and activates GSK3B kinase activity. Then, GSK3B phosphorylates ETS1 and activates its activity. | [17], [18], [19]{Liu, 2004 #725} |
| MYL2 to ACTC1 | N/A |  |
| MYLK to PRKACB | MYLK1 phosphorylayes FAK2. FAK2 activates PDK, then promotes PRKACB activity. | [20], [21], [22] |
| PIK3CB to LYN | PIK3CB phosphorylates AKT1 and increases its activity. Then, AKT phosphorylates RARalpha which directly regulates Lyn expression. | [23], [24], [25], [26] |
| PIK3CG to ICOS | PIK3CG physically interacts with PKC-zeta and increases its activity. Then, PKC-zeta phosphorylates NFATC2 to activate ICOS in the transcription level. | [27], [28], [29], [30], [31] |
| PREX1 to PIK3R5 | PREX1 induce Rac1 activation.  Rac1 B further activates PKC-alpha to phosphorylates G-protein gamma 12. Then, PIK3R5 activity will be reduced eventually. | [32], [33], [34], [35], [36], [13], [37] |
| PXN to PTK2 | PXN physically interacts with PTK2 and increases its activity | [38] [39] [40] |
| RAP1A to PRKACB | RAP-1A physically interacts with Tiam2 and increases its activity. Next, Tiam2 activates Rac1, thereby promoting MYC expression that further enhances PKA-cat beta expression. | [41], [42], [43], [44], [45], [46] |

1. Xu, Y.Z., et al., *Nuclear translocation of beta-actin is involved in transcriptional regulation during macrophage differentiation of HL-60 cells.* Mol Biol Cell, 2010. **21**(5): p. 811-20.

2. Qin, L., et al., *Steroid receptor coactivator-1 upregulates integrin alpha expression to promote breast cancer cell adhesion and migration.* Cancer Res, 2011. **71**(5): p. 1742-51.

3. Dunlap, K., J.I. Luebke, and T.J. Turner, *Exocytotic Ca2+ channels in mammalian central neurons.* Trends Neurosci, 1995. **18**(2): p. 89-98.

4. Lehmann-Horn, F. and K. Jurkat-Rott, *Voltage-gated ion channels and hereditary disease.* Physiol Rev, 1999. **79**(4): p. 1317-72.

5. Basu, A. and U. Sivaprasad, *Protein kinase Cepsilon makes the life and death decision.* Cell Signal, 2007. **19**(8): p. 1633-42.

6. Iannazzo, L., *Involvement of B-50 (GAP-43) phosphorylation in the modulation of transmitter release by protein kinase C.* Clin Exp Pharmacol Physiol, 2001. **28**(11): p. 901-4.

7. Liu, W.S. and C.A. Heckman, *The sevenfold way of PKC regulation.* Cell Signal, 1998. **10**(8): p. 529-42.

8. Samanna, V., et al., *Alpha-V-dependent outside-in signaling is required for the regulation of CD44 surface expression, MMP-2 secretion, and cell migration by osteopontin in human melanoma cells.* Exp Cell Res, 2006. **312**(12): p. 2214-30.

9. Giannelli, G., et al., *Induction of cell migration by matrix metalloprotease-2 cleavage of laminin-5.* Science, 1997. **277**(5323): p. 225-8.

10. McDonald, P.C., A.B. Fielding, and S. Dedhar, *Integrin-linked kinase--essential roles in physiology and cancer biology.* J Cell Sci, 2008. **121**(Pt 19): p. 3121-32.

11. Martin, M.B., et al., *A role for Akt in mediating the estrogenic functions of epidermal growth factor and insulin-like growth factor I.* Endocrinology, 2000. **141**(12): p. 4503-11.

12. Levy, N., et al., *Differential regulation of native estrogen receptor-regulatory elements by estradiol, tamoxifen, and raloxifene.* Mol Endocrinol, 2008. **22**(2): p. 287-303.

13. Brock, C., et al., *Roles of G beta gamma in membrane recruitment and activation of p110 gamma/p101 phosphoinositide 3-kinase gamma.* J Cell Biol, 2003. **160**(1): p. 89-99.

14. Hess, F., et al., *Integrin-linked kinase interacts with caspase-9 and -8 in an adhesion-dependent manner for promoting radiation-induced apoptosis in human leukemia cells.* Oncogene, 2007. **26**(10): p. 1372-84.

15. Senft, J., B. Helfer, and S.M. Frisch, *Caspase-8 interacts with the p85 subunit of phosphatidylinositol 3-kinase to regulate cell adhesion and motility.* Cancer Res, 2007. **67**(24): p. 11505-9.

16. Meier, T.I., et al., *Cloning, expression, purification, and characterization of the human Class Ia phosphoinositide 3-kinase isoforms.* Protein Expr Purif, 2004. **35**(2): p. 218-24.

17. Kandasamy, A.D. and R. Schulz, *Glycogen synthase kinase-3beta is activated by matrix metalloproteinase-2 mediated proteolysis in cardiomyoblasts.* Cardiovasc Res, 2009. **83**(4): p. 698-706.

18. Liu, H., et al., *AML1/Runx1 recruits calcineurin to regulate granulocyte macrophage colony-stimulating factor by Ets1 activation.* J Biol Chem, 2004. **279**(28): p. 29398-408.

19. Linding, R., et al., *Systematic discovery of in vivo phosphorylation networks.* Cell, 2007. **129**(7): p. 1415-26.

20. Xu, J., et al., *Nonmuscle myosin light-chain kinase mediates neutrophil transmigration in sepsis-induced lung inflammation by activating beta2 integrins.* Nat Immunol, 2008. **9**(8): p. 880-6.

21. Taniyama, Y., et al., *Pyk2- and Src-dependent tyrosine phosphorylation of PDK1 regulates focal adhesions.* Mol Cell Biol, 2003. **23**(22): p. 8019-29.

22. Nirula, A., et al., *Phosphoinositide-dependent kinase 1 targets protein kinase A in a pathway that regulates interleukin 4.* J Exp Med, 2006. **203**(7): p. 1733-44.

23. Su, C.H., et al., *Akt phosphorylation at Thr308 and Ser473 is required for CHIP-mediated ubiquitination of the kinase.* Cell Signal, 2011. **23**(11): p. 1824-30.

24. Jones, N., et al., *Tie receptors: new modulators of angiogenic and lymphangiogenic responses.* Nat Rev Mol Cell Biol, 2001. **2**(4): p. 257-67.

25. Srinivas, H., et al., *Akt phosphorylates and suppresses the transactivation of retinoic acid receptor alpha.* Biochem J, 2006. **395**(3): p. 653-62.

26. Rice, K.L., et al., *Comprehensive genomic screens identify a role for PLZF-RARalpha as a positive regulator of cell proliferation via direct regulation of c-MYC.* Blood, 2009. **114**(27): p. 5499-511.

27. Frey, R.S., et al., *Phosphatidylinositol 3-kinase gamma signaling through protein kinase Czeta induces NADPH oxidase-mediated oxidant generation and NF-kappaB activation in endothelial cells.* J Biol Chem, 2006. **281**(23): p. 16128-38.

28. Lehmann, K., et al., *PI3Kgamma controls oxidative bursts in neutrophils via interactions with PKCalpha and p47phox.* Biochem J, 2009. **419**(3): p. 603-10.

29. Gomez-Casero, E., et al., *Cot/Tpl2 and PKCzeta cooperate in the regulation of the transcriptional activity of NFATc2 through the phosphorylation of its amino-terminal domain.* Cell Signal, 2007. **19**(8): p. 1652-61.

30. San-Antonio, B., M.A. Iniguez, and M. Fresno, *Protein kinase Czeta phosphorylates nuclear factor of activated T cells and regulates its transactivating activity.* J Biol Chem, 2002. **277**(30): p. 27073-80.

31. Tan, A.H., et al., *T helper cell-specific regulation of inducible costimulator expression via distinct mechanisms mediated by T-bet and GATA-3.* J Biol Chem, 2008. **283**(1): p. 128-36.

32. Wang, Z., et al., *Lack of a significant role of P-Rex1, a major regulator of macrophage Rac1 activation and chemotaxis, in atherogenesis.* Prostaglandins Other Lipid Mediat, 2008. **87**(1-4): p. 9-13.

33. Sosa, M.S., et al., *Identification of the Rac-GEF P-Rex1 as an essential mediator of ErbB signaling in breast cancer.* Mol Cell, 2010. **40**(6): p. 877-92.

34. Urano, D., et al., *Domain-domain interaction of P-Rex1 is essential for the activation and inhibition by G protein betagamma subunits and PKA.* Cell Signal, 2008. **20**(8): p. 1545-54.

35. Slater, S.J., et al., *Interaction of protein kinase C isozymes with Rho GTPases.* Biochemistry, 2001. **40**(14): p. 4437-45.

36. Yasuda, H., et al., *Phosphorylation of the G protein gamma12 subunit regulates effector specificity.* J Biol Chem, 1998. **273**(34): p. 21958-65.

37. Rommel, C., M. Camps, and H. Ji, *PI3K delta and PI3K gamma: partners in crime in inflammation in rheumatoid arthritis and beyond?* Nat Rev Immunol, 2007. **7**(3): p. 191-201.

38. Moeller, M.L., et al., *EphB receptors regulate dendritic spine morphogenesis through the recruitment/phosphorylation of focal adhesion kinase and RhoA activation.* J Biol Chem, 2006. **281**(3): p. 1587-98.

39. Lyons, P.D., et al., *Inhibition of the catalytic activity of cell adhesion kinase beta by protein-tyrosine phosphatase-PEST-mediated dephosphorylation.* J Biol Chem, 2001. **276**(26): p. 24422-31.

40. Turner, C.E., et al., *Paxillin LD4 motif binds PAK and PIX through a novel 95-kD ankyrin repeat, ARF-GAP protein: A role in cytoskeletal remodeling.* J Cell Biol, 1999. **145**(4): p. 851-63.

41. Zaldua, N., et al., *Epac signaling pathway involves STEF, a guanine nucleotide exchange factor for Rac, to regulate APP processing.* FEBS Lett, 2007. **581**(30): p. 5814-8.

42. Yamauchi, J., et al., *The neurotrophin-3 receptor TrkC directly phosphorylates and activates the nucleotide exchange factor Dbs to enhance Schwann cell migration.* Proc Natl Acad Sci U S A, 2005. **102**(14): p. 5198-203.

43. Buongiorno, P., et al., *Rac1 GTPase and the Rac1 exchange factor Tiam1 associate with Wnt-responsive promoters to enhance beta-catenin/TCF-dependent transcription in colorectal cancer cells.* Mol Cancer, 2008. **7**: p. 73.

44. Zeller, K.I., et al., *Global mapping of c-Myc binding sites and target gene networks in human B cells.* Proc Natl Acad Sci U S A, 2006. **103**(47): p. 17834-9.

45. Margolin, A.A., et al., *ChIP-on-chip significance analysis reveals large-scale binding and regulation by human transcription factor oncogenes.* Proc Natl Acad Sci U S A, 2009. **106**(1): p. 244-9.

46. Lin, C.H., et al., *Gene regulation and epigenetic remodeling in murine embryonic stem cells by c-Myc.* PLoS One, 2009. **4**(11): p. e7839.

Supplementary Table S2. The result of the multi-way ANOVA for identification of critical factors affecting causal network inference

|  | **Df** | **Sum Sq** | **Mean Sq** | **F value** | **Pr(>F)** |
| --- | --- | --- | --- | --- | --- |
| R | 1 | 0.00172 | 0.00172 | 0.867 | 0.371825 |
| K | 1 | 0.0011 | 0.0011 | 0.554 | 0.472475 |
| SNP | 1 | 0.05491 | 0.05491 | 27.651 | 0.000269 |
| Merge | 1 | 0.00207 | 0.00207 | 1.045 | 0.328677 |
| Residuals | 11 | 0.02184 | 0.00199 |  |  |

Supplementary Figure S1. Different module size affects the time complexity of constructing a Bayesian network for each pair of modules. (a) The running time grows exponentially when the module size expands. The time required increases rapidly as module size is large than 15. Besides, the time needed for module size of 15 is almost twofold than that for module size of 10. Accordingly, we set the maximal module size to 10 for efficient subnetwork inference. (b) Different module size does not affect the final inferred network too much. The concordance rate fluctuates slightly with the change of module size.


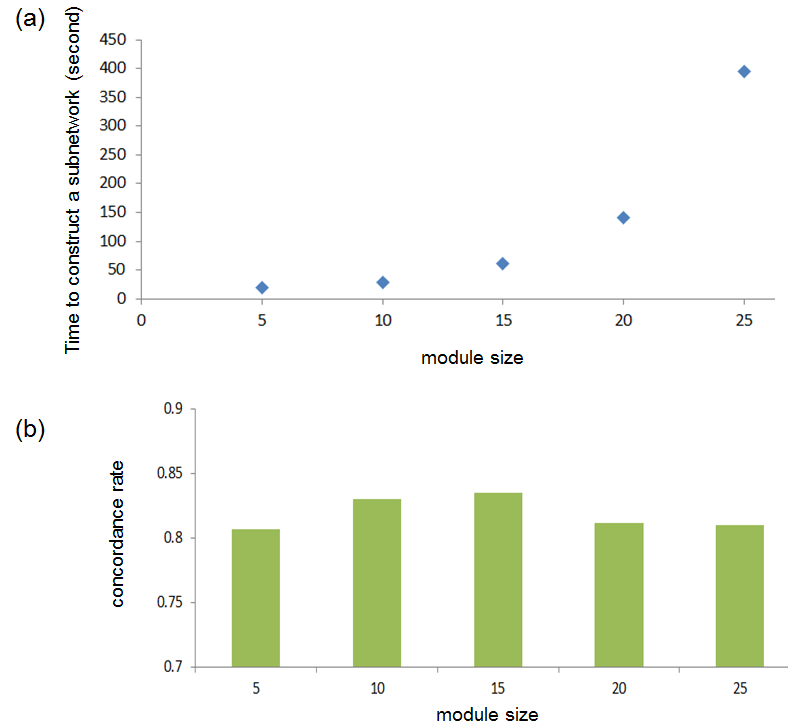


Supplementary Figure S2. Receiver operator characteristic analysis for the six-gene model (MDH2, RXRB, FABP1, ACADM, APOA5, and PPARG). ROC analysis indicates that this predictive model has an area under the curve (AUC) of 0.925 and a favorable sensitivity and specificity of 0.921 and 0.731, respectively.


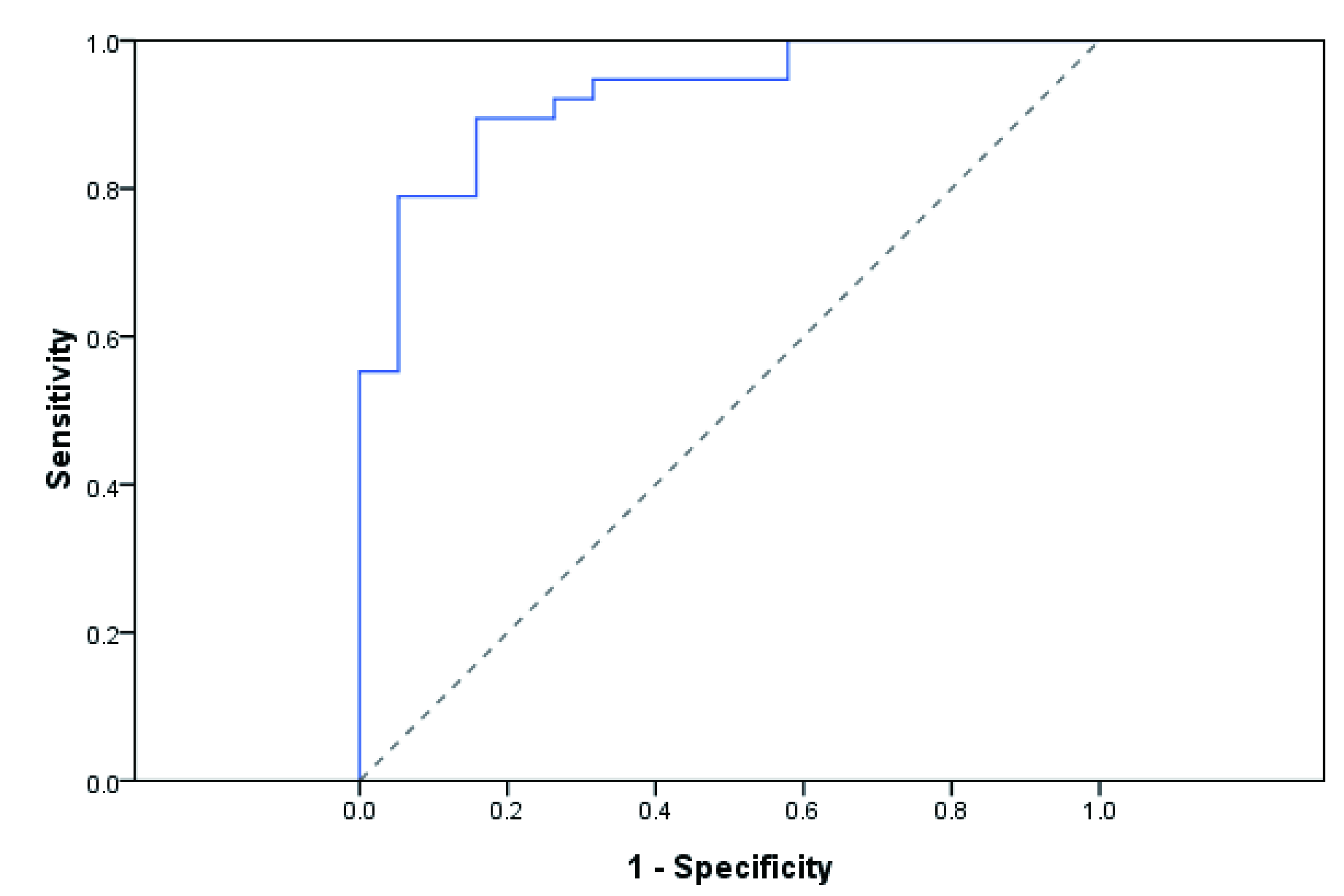

Supplement: Supplementary Data [file supp_gkt1277_nar-02423-z-2013-File008.docx]
